# Correction: Visualizing the structure of RNA-seq expression data using grade of membership models

**DOI:** 10.1371/journal.pgen.1006759

**Published:** 2017-05-26

**Authors:** Kushal K. Dey, Chiaowen Joyce Hsiao, Matthew Stephens

## Notice of Republication

This article was republished on April 11, 2017, to replace Fig 2. An incorrect figure was typeset as Fig 2 during the production process. Please download this article again to view the correct version, with the correct Fig 2 included. The originally published, uncorrected article and the republished, corrected articles are provided here for reference.

## Supporting information

S1 File Originally published, uncorrected article. (PDF)

S2 File Republished, corrected article. (PDF)
