# Supplementary material for: Visualizing the structure of RNA-seq expression data using grade of membership models
Source: PLoS Genet. 2017 Mar 23;13(3):e1006599. doi: 10.1371/journal.pgen.1006599 (PMC5363805; doi:10.1371/journal.pgen.1006599)
Supplement: S1 Fig — Some tissues form a separate cluster from the other tissues from K = 5 onwards (for example: Whole Blood, Skin), whereas some tissue only form a distinctive subgroup at K = 20 (for example: Arteries). (PDF) [file pgen.1006599.s001.pdf]

S1 Fig. Structure plot of GTEx V6 tissue samples for (A) $K = 5$**, (B)** $K = 10$**, (C)** $K = 15$**, (D)** $K = 20$**.** Some tissues form a separate cluster from the other tissues from $K = 5$ onwards (for example: Whole Blood, Skin), whereas some tissue only form a distinctive subgroup at $K = 20$ (for example: Arteries).

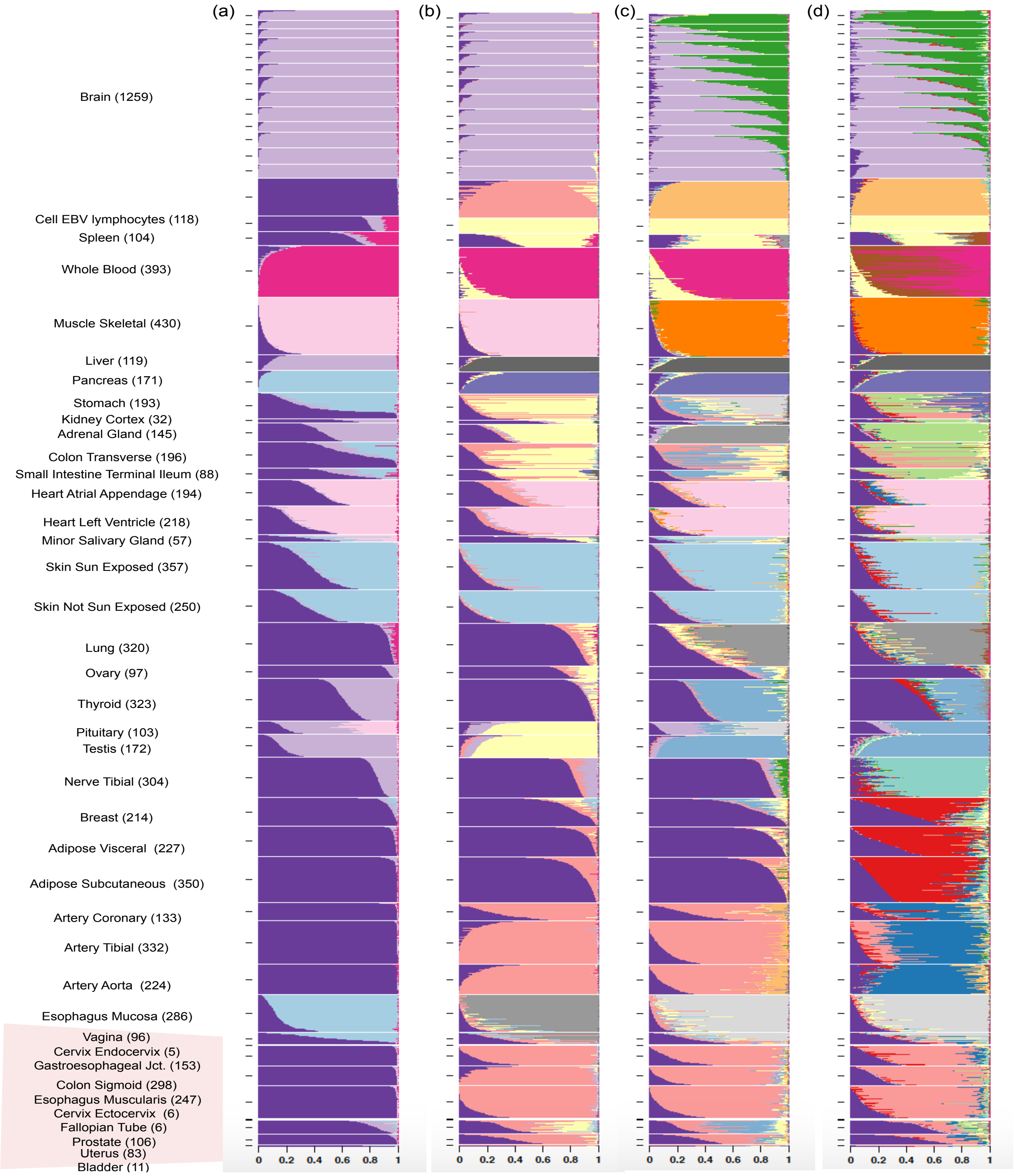

(a)
(b)
(c)
(d)
Brain (1259)
Cell EBV lymphocytes (118)
Spleen (104)
Whole Blood (393)
Muscle Skeletal (430)
Liver (119)
Pancreas (171)
Stomach (193)
Kidney Cortex (32)
Adrenal Gland (145)
Colon Transverse (196)
Small Intestine Terminal Ileum (88)
Heart Atrial Appendage (194)
Heart Left Ventricle (218)
Minor Salivary Gland (57)
Skin Sun Exposed (357)
Skin Not Sun Exposed (250)
Lung (320)
Ovary (97)
Thyroid (323)
Pituitary (103)
Testis (172)
Nerve Tibial (304)
Breast (214)
Adipose Visceral (227)
Adipose Subcutaneous (350)
Artery Coronary (133)
Artery Tibial (332)
Artery Aorta (224)
Esophagus Mucosa (286)
Vagina (96)
Cervix Endocervix (5)
Gastroesophageal Jct. (153)
Colon Sigmoid (298)
Esophagus Muscularis (247)
Cervix Ectocervix (6)
Fallopian Tube (6)
Prostate (106)
Uterus (83)
Bladder (11)
0 0.2 0.4 0.6 0.8 1
