# Supplementary material for: Visualizing the structure of RNA-seq expression data using grade of membership models
Source: PLoS Genet. 2017 Mar 23;13(3):e1006599. doi: 10.1371/journal.pgen.1006599 (PMC5363805; doi:10.1371/journal.pgen.1006599)

S2 Fig. Top five principal components (PC) for GTEx V6 tissue samples. Scatter plot representation of the top five PCs of the GTEx tissue samples. Data was transformed to log2 counts per million (CPM).

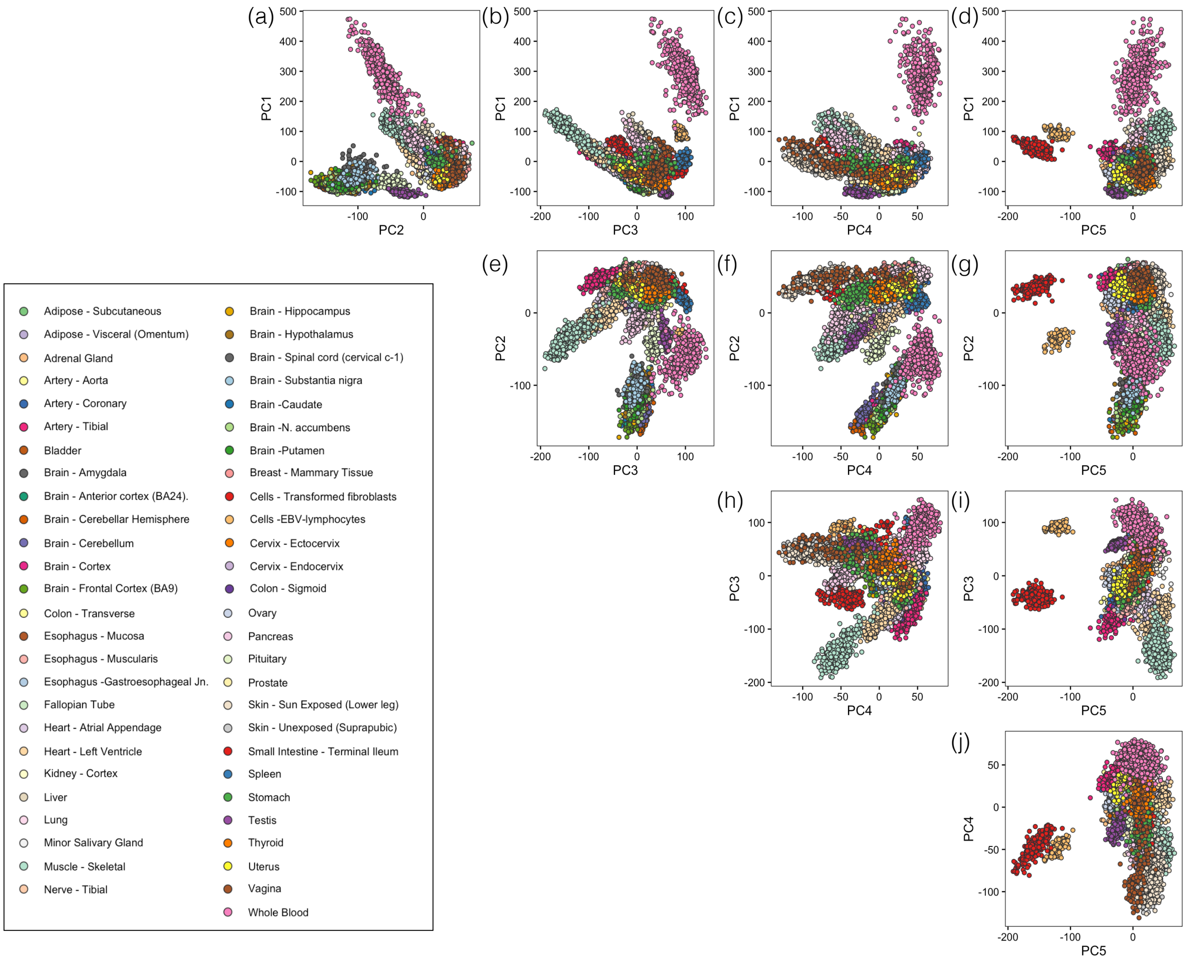

Supplement: S2 Fig — Scatter plot representation of the top five PCs of the GTEx tissue samples. Data was transformed to log2 counts per million (CPM). (PDF) [file pgen.1006599.s002.pdf]
