# Supplementary material for: Visualizing the structure of RNA-seq expression data using grade of membership models
Source: PLoS Genet. 2017 Mar 23;13(3):e1006599. doi: 10.1371/journal.pgen.1006599 (PMC5363805; doi:10.1371/journal.pgen.1006599)
Supplement: S3 Fig — We used GTEx V6 data for model performance comparisons. Specifically, for every pair of the 53 tissues, we assessed the ability of the methods to separate samples according to their tissue of origin. The subplots of heatmaps show the results of evaluation under different scenarios. Filled squares in the heatmap indicate successful separation of the samples in corresponding tissue pair comparison. (a) Hierarchical clustering on log2 counts per million (CPM) transformed data using Euclidean distance. (b) Hierarchical clustering on the standardized log2-CPM transformed data (transformed values for each gene was mean and scale transformed) using the Euclidean distance. (c) GoM model of K = 2 applied to counts. (d) Hierarchical clustering on counts data with the assumption that, for each gene the sample read count cng has a variance c¯g+1 that is constant across samples. And, the the gene-specific variance c¯g+1 was used to scale the distance matrix for clustering. (e) Hierarchical clustering applied to adjusted count data. Each gene has a mean expression value of 0 and variance of 1. Taken together, these results suggest that regardless of the different data transformation scenarios, the GoM model with K = 2 is able to separate samples of different tissue of origin, better than hierarchical cluster methods. (PDF) [file pgen.1006599.s003.pdf]

S3 Fig. Comparison between GoM model and hierarchical clustering under different scenarios of data transformation. We used GTEx V6 data for model performance comparisons. Specifically, for every pair of the 53 tissues, we assessed the ability of the methods to separate samples according to their tissue of origin. The subplots of heatmaps show the results of evaluation under different scenarios. Filled squares in the heatmap indicate successful separation of the samples in corresponding tissue pair comparison. (a) Hierarchical clustering on log2 counts per million (CPM) transformed data using Euclidean distance. (b) Hierarchical clustering on the standardized log2-CPM transformed data (transformed values for each gene was mean and scale transformed) using the Euclidean distance. (c) GoM model of $K = 2$ applied to counts.(d) Hierarchical clustering on counts data with the assumption that, for each gene the sample read count c_{ng} has a variance $\bar{c}_g + 1$ that is constant across samples. And, the the gene-specific variance $\bar{c}_g + 1$ was used to scale the distance matrix for clustering. (e) Hierarchical clustering applied to adjusted count data from (c). Each gene expression is further normalized over (c) to have mean expression value of 0 and variance of 1. Taken together, these results suggest that regardless of the different data transformation scenarios, the GoM model with $K = 2$ is able to separate samples of different tissue of origin, better than hierarchical cluster methods.

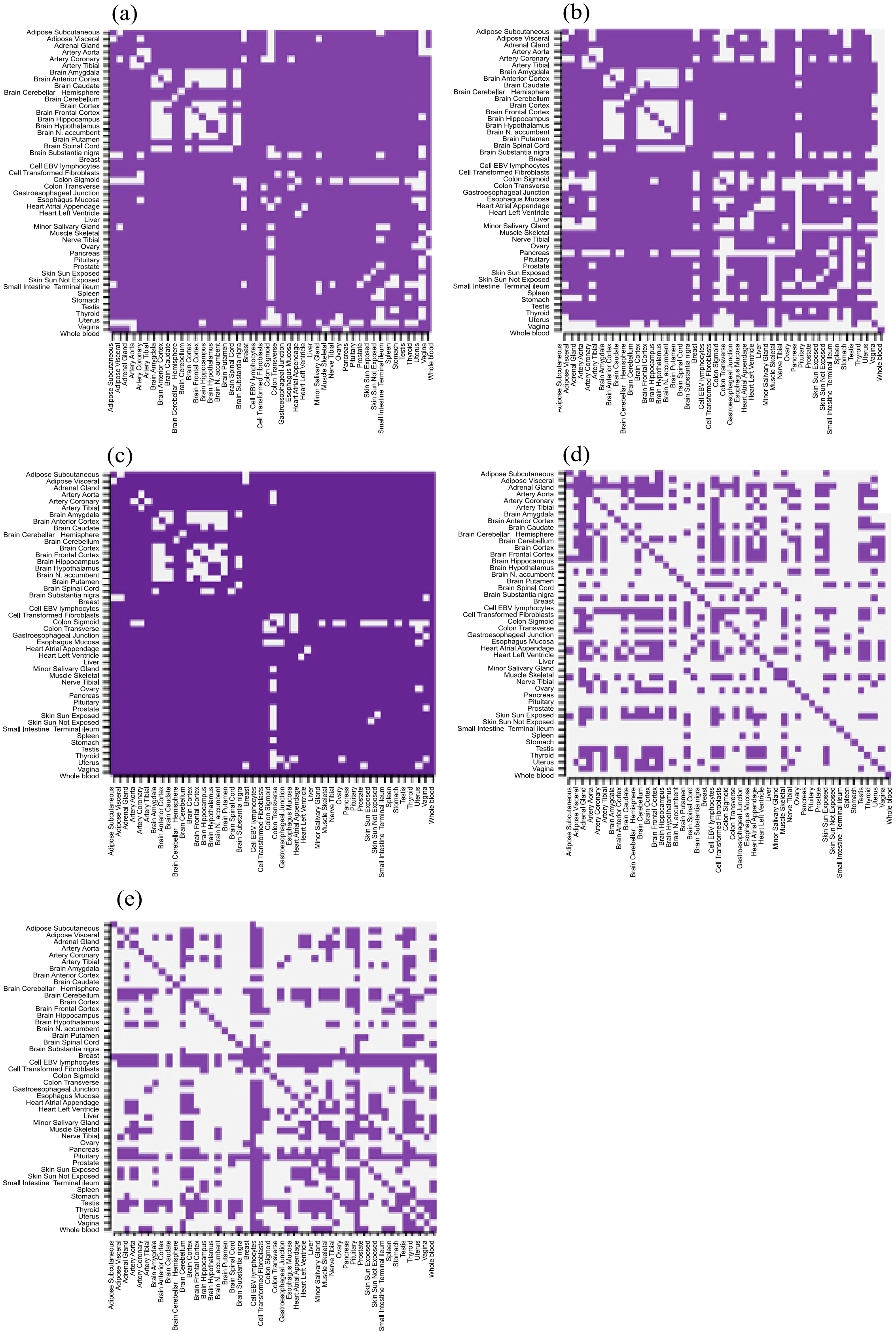
(a)
(b)
(c)
(d)
(e)
Adipose Subcutaneous
Adipose Visceral
Adrenal Gland
Artery Aorta
Artery Coronary
Artery Tibial
Brain Amygdala
Brain Anterior Cortex
Brain Caudate
Brain Cerebellar Hemisphere
Brain Cerebellum
Brain Cortex
Brain Frontal Cortex
Brain Hippocampus
Brain Hypothalamus
Brain N. accumbent
Brain Putamen
Brain Spinal Cord
Brain Substantia nigra
Breast
Cell EBV lymphocytes
Cell Transformed Fibroblasts
Colon Sigmoid
Colon Transverse
Gastroesophageal Junction
Esophagus Mucosa
Heart Atrial Appendage
Heart Left Ventricle
Liver
Minor Salivary Gland
Muscle Skeletal
Nerve Tibial
Ovary
Pancreas
Pituitary
Prostate
Skin Sun Exposed
Skin Sun Not Exposed
Small Intestine Terminal ileum
Spleen
Stomach
Testis
Thyroid
Uterus
Vagina
Whole blood
