# Supplementary material for: Visualizing the structure of RNA-seq expression data using grade of membership models
Source: PLoS Genet. 2017 Mar 23;13(3):e1006599. doi: 10.1371/journal.pgen.1006599 (PMC5363805; doi:10.1371/journal.pgen.1006599)

S4 Fig. GTEx brain tissue samples visualization using (a) principle component analysis, (b) t-SNE, and (c) Multidimensional scaling and (d) dendrogram for hierarchical clustering. The colors represent the 13 different brain tissue types. In (a) and (b), the majority of the tissue samples are distinct from Cerebellum tissue samples (the cluster of samples located on the right side of the plot). While, in (c), most tissue samples are located at the enter of the plot and are similar to each other in the t-SNE dimensions. In (d), samples from Brain Cerebellar, Cerebellar Hemisphere seem to cluster together and separate from samples from other brain regions. But, because of the large number of samples, patterns of variation between tissue samples are difficult to detect.

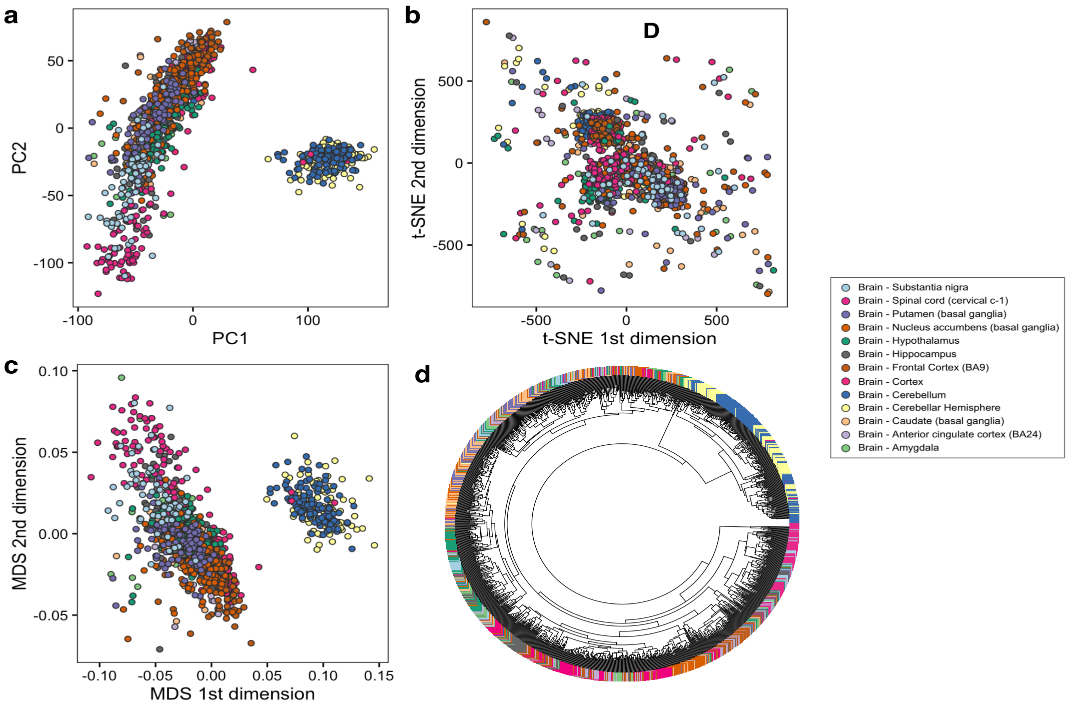

Supplement: S4 Fig — (PDF) [file pgen.1006599.s004.pdf]
