# Supplementary material for: Visualizing the structure of RNA-seq expression data using grade of membership models
Source: PLoS Genet. 2017 Mar 23;13(3):e1006599. doi: 10.1371/journal.pgen.1006599 (PMC5363805; doi:10.1371/journal.pgen.1006599)
Supplement: S5 Fig — (a) pthin = 0.01 and (B) pthin = 0.0001. The structure in these two plots closely resemble the pattern observed in Fig 1(a), though there are a few differences from the unthinned version. (PDF) [file pgen.1006599.s005.pdf]

S5 Fig. Structure plot of GTEx V6 tissue samples for $K = 20$ in two runs under different thinning parameter settings. (a) $p_{thin} = 0.01$ and (b) $p_{thin} = 0.0001$. The structural patterns in these two plots closely resemble the structural patterns in Fig 1(a), though there are a few differences from the unthinned version.

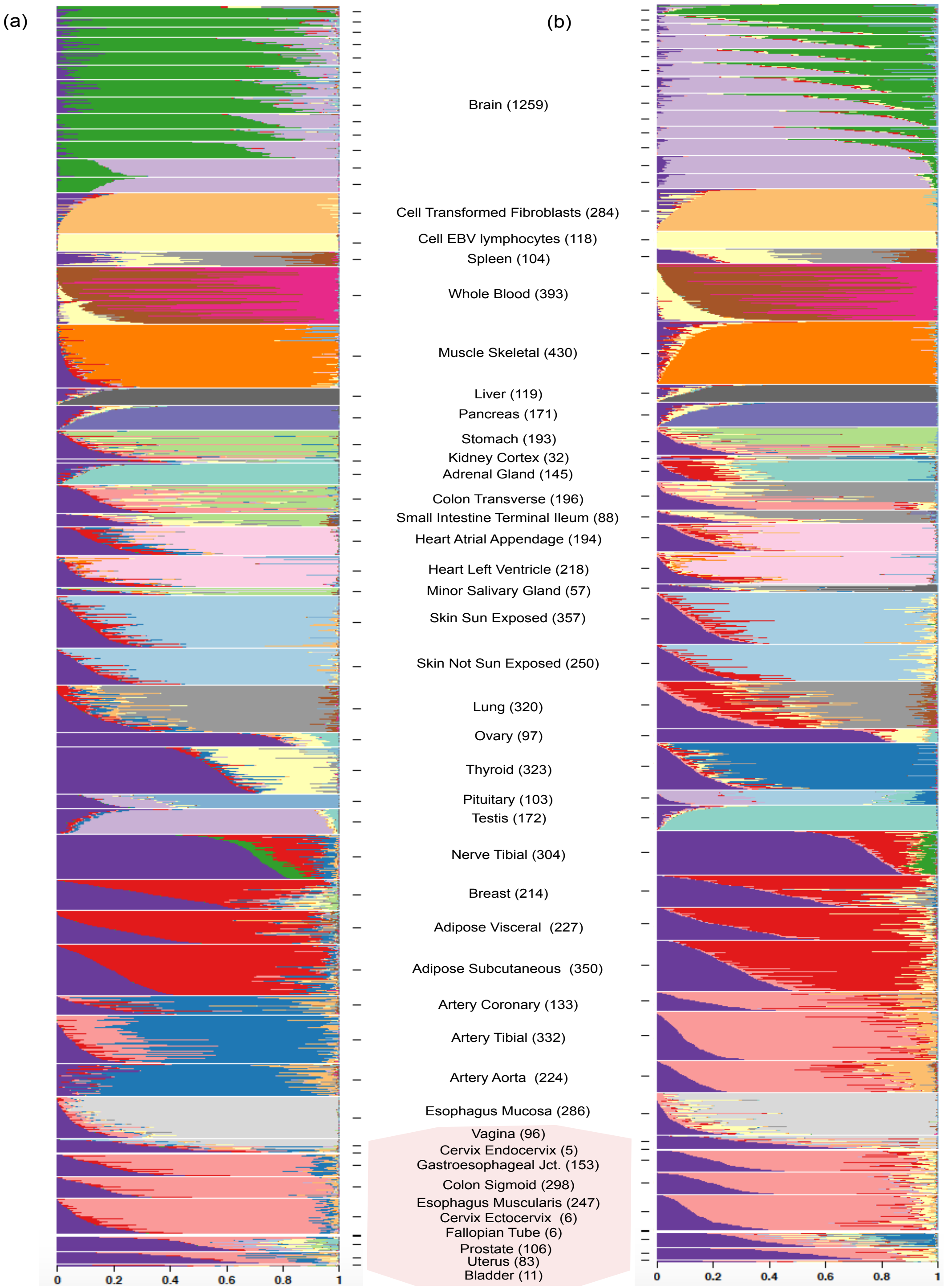
(a)
(b)
Brain (1259)
Cell Transformed Fibroblasts (284)
Cell EBV lymphocytes (118)
Spleen (104)
Whole Blood (393)
Muscle Skeletal (430)
Liver (119)
Pancreas (171)
Stomach (193)
Kidney Cortex (32)
Adrenal Gland (145)
Colon Transverse (196)
Small Intestine Terminal Ileum (88)
Heart Atrial Appendage (194)
Heart Left Ventricle (218)
Minor Salivary Gland (57)
Skin Sun Exposed (357)
Skin Not Sun Exposed (250)
Lung (320)
Ovary (97)
Thyroid (323)
Pituitary (103)
Testis (172)
Nerve Tibial (304)
Breast (214)
Adipose Visceral (227)
Adipose Subcutaneous (350)
Artery Coronary (133)
Artery Tibial (332)
Artery Aorta (224)
Esophagus Mucosa (286)
Vagina (96)
Cervix Endocervix (5)
Gastroesophageal Jct. (153)
Colon Sigmoid (298)
Esophagus Muscularis (247)
Cervix Ectocervix (6)
Fallopian Tube (6)
Prostate (106)
Uterus (83)
Bladder (11)
0
0.2
0.4
0.6
0.8
1
