# Supplementary material for: Visualizing the structure of RNA-seq expression data using grade of membership models
Source: PLoS Genet. 2017 Mar 23;13(3):e1006599. doi: 10.1371/journal.pgen.1006599 (PMC5363805; doi:10.1371/journal.pgen.1006599)
Supplement: S6 Fig — For each pair of tissue samples from the GTEx V6 data we assessed whether or not each clustering method (with K = 2 clusters) separated the samples according to their tissue of origin, with successful separation indicated by a filled square. Thinning deteriorates accuracy compared with the unthinned data (Fig 2), but even then the model-based method remains more successful than the hierarchical clustering in separating the samples by tissue or origin. (PDF) [file pgen.1006599.s006.pdf]

S6 Fig. A comparison of "accuracy" of hierarchical clustering vs. GoM on thinned GTEx data, with thinning parameters of $p_{thin} = 0.01$ and $p_{thin} = 0.001$. For each pair of tissue samples from the GTEx V6 data we assessed whether or not each clustering method (with $K = 2$ clusters) separated the samples according to their tissue of origin, with successful separation indicated by a filled square. Thinning deteriorates accuracy compared with the unthinned data (Fig 2), but even then the model-based method remains more successful than the hierarchical clustering in separating the samples by tissue or origin.

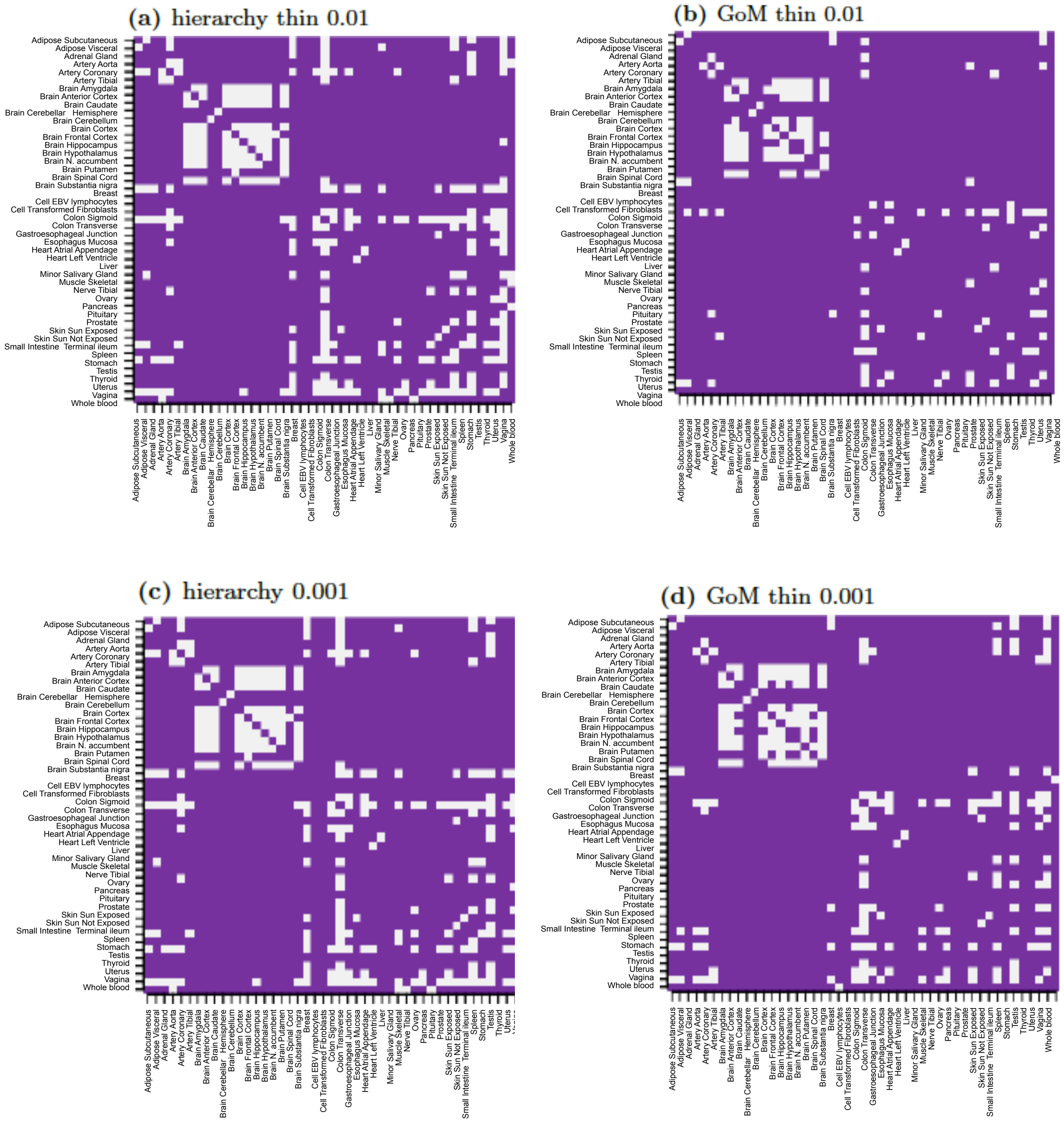
(a) hierarchy thin 0.01
(b) GoM thin 0.01
(c) hierarchy 0.001
(d) GoM thin 0.001
Adipose Subcutaneous
Adipose Visceral
Adrenal Gland
Artery Aorta
Artery Coronary
Artery Tibial
Brain Amygdala
Brain Anterior Cortex
Brain Caudate
Brain Cerebellar Hemisphere
Brain Cerebellum
Brain Cortex
Brain Frontal Cortex
Brain Hippocampus
Brain Hypothalamus
Brain N. accumbent
Brain Putamen
Brain Spinal Cord
Brain Substantia nigra
Breast
Cell EBV lymphocytes
Cell Transformed Fibroblasts
Colon Sigmoid
Colon Transverse
Gastroesophageal Junction
Esophagus Mucosa
Heart Atrial Appendage
Heart Left Ventricle
Liver
Minor Salivary Gland
Muscle Skeletal
Nerve Tibial
Ovary
Pancreas
Pituitary
Prostate
Skin Sun Exposed
Skin Sun Not Exposed
Small Intestine Terminal ileum
Spleen
Stomach
Testis
Thyroid
Uterus
Vagina
Whole blood
