# Supplementary material for: Visualizing the structure of RNA-seq expression data using grade of membership models
Source: PLoS Genet. 2017 Mar 23;13(3):e1006599. doi: 10.1371/journal.pgen.1006599 (PMC5363805; doi:10.1371/journal.pgen.1006599)

S7 Fig. Visualizing mouse pre-implantation embryos data from Deng et al (2014) using (a) Principle Component Analysis, (b) t-SNE, (c) Multidimensional Scaling (MDS) and (d) circular dendrogram for hierarchical clustering. The colors represent different developmental stages. PCA, MDS seem to be effective in capturing the developmental trajectory, but t-SNE fails to do so. Hierarchical clustering fails to separate out the blastocyst cells from the cells in early stages of development completely.

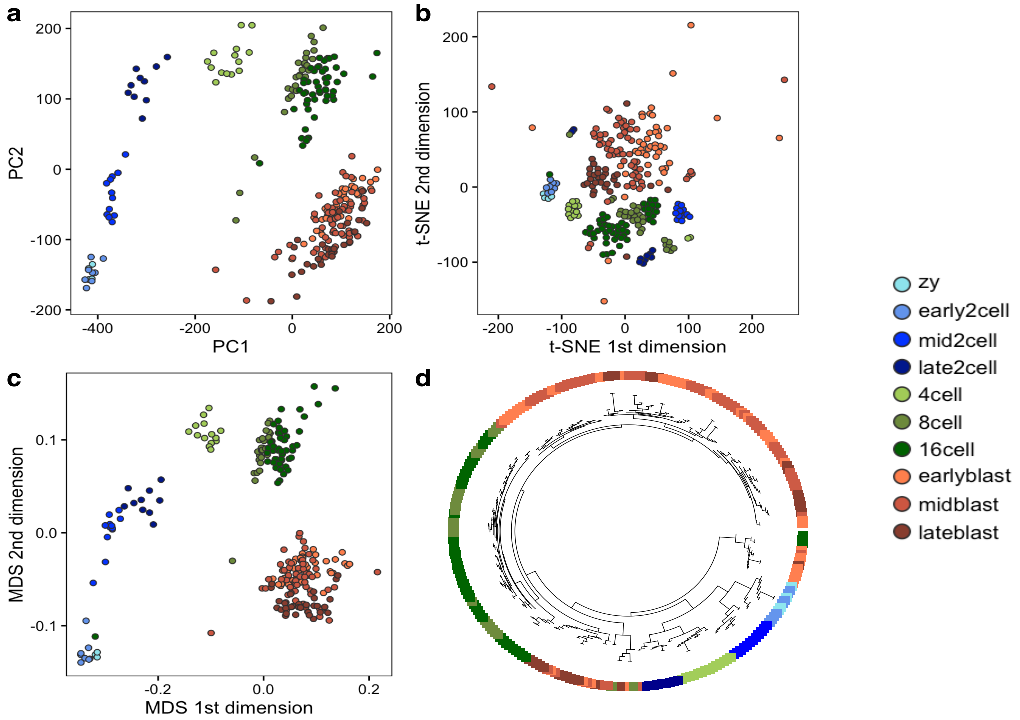

Supplement: S7 Fig — (PDF) [file pgen.1006599.s007.pdf]
