# Supplementary material for: Visualizing the structure of RNA-seq expression data using grade of membership models
Source: PLoS Genet. 2017 Mar 23;13(3):e1006599. doi: 10.1371/journal.pgen.1006599 (PMC5363805; doi:10.1371/journal.pgen.1006599)

S8 Fig. Additional GoM analysis of Deng et al (2014) data including blastocyst samples and 48 blastocyst marker genes. We considered 48 blastocyst marker genes (as chosen by Guo et al., 2010) and fitted GoM model with $K = 3$ to 133 blastocyst samples. In the Structure plot, blastocyst samples are arranged in order of estimated membership proportion in the Green cluster. The panel located above the Structure plot shows the corresponding pre-implantation stage from which blastocyst samples were collected. The heatmap located below the Structure plot represents expression levels of the 48 blastocyst marker genes (log2 CPM), and the corresponding dendrogram shows results of hierarchical clustering (complete linkage). The table on the right of the expression heatmap displays gene information, showing, from left to right, 1) whether or not the gene is a transcription factor, 2) the driving GoM cluster if the gene was among the top five driving genes, and 3) the featured cell type (TE: trophecoderm, EPI: epiblast, PE: primitive endoderm) that was found in Guo et al., 2010.

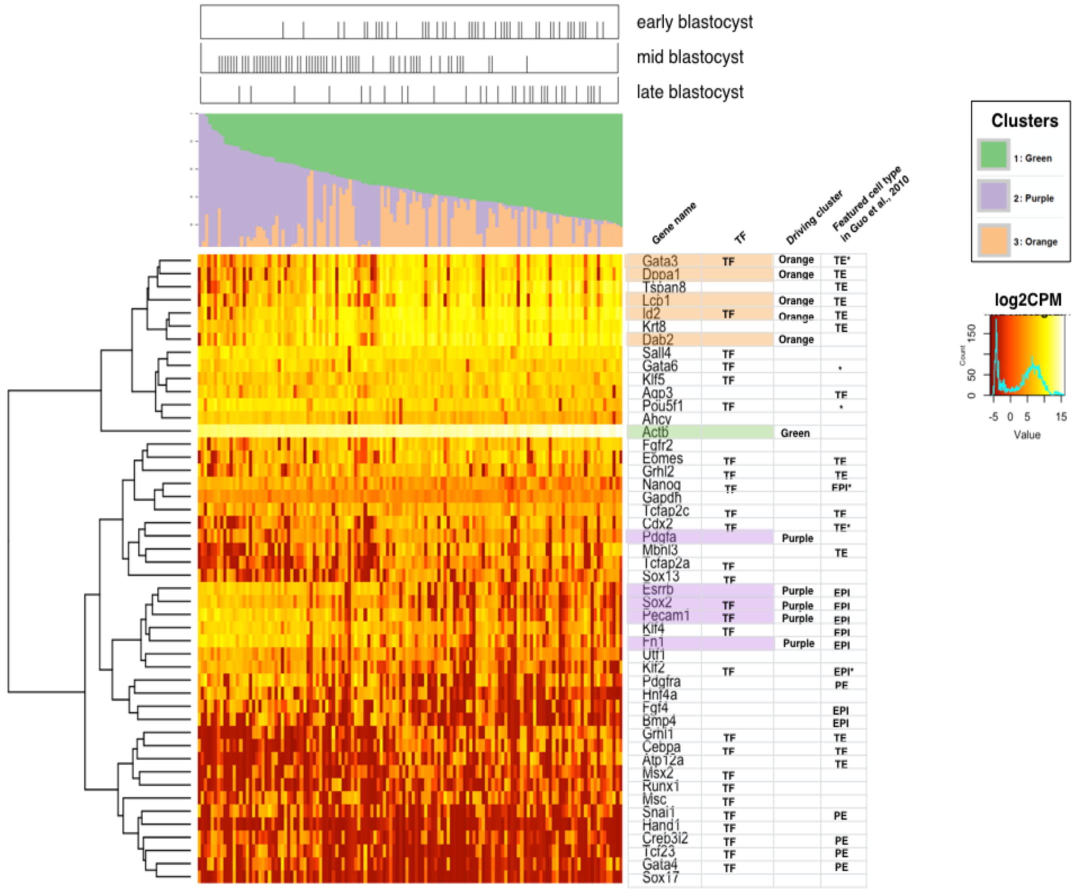

Supplement: S8 Fig — We considered 48 blastocyst marker genes (as chosen by Guo et al., 2010) and fitted GoM model with K = 3 to 133 blastocyst samples. In the Structure plot, blastocyst samples are arranged in order of estimated membership proportion in the Green cluster. The panel located above the Structure plot shows the corresponding pre-implantation stage from which blastocyst samples were collected. The heatmap located below the Structure plot represents expression levels of the 48 blastocyst marker genes (log2 CPM), and the corresponding dendrogram shows results of hierarchical clustering (complete linkage). The table on the right of the expression heatmap displays gene information, showing, from left to right, 1) whether or not the gene is a transcription factor, 2) the driving GoM cluster if the gene was among the top five driving genes, and 3) the featured cell type (TE: trophecoderm, EPI: epiblast, PE: primitive endoderm) that was found in Guo et al., 2010. (PDF) [file pgen.1006599.s008.pdf]
