# Supplementary material for: Visualizing the structure of RNA-seq expression data using grade of membership models
Source: PLoS Genet. 2017 Mar 23;13(3):e1006599. doi: 10.1371/journal.pgen.1006599 (PMC5363805; doi:10.1371/journal.pgen.1006599)

S9 Fig. Visualization of PCA and t-SNE results of mouse pre-implantation embryos data from Deng et al (2014) using 48 blastocyst marker genes

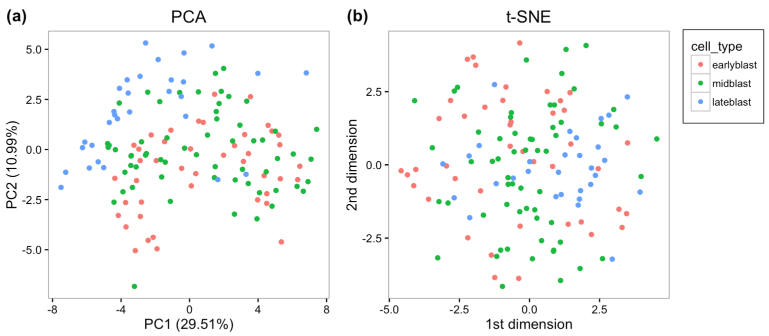

Supplement: S9 Fig — (PDF) [file pgen.1006599.s009.pdf]
