# Supplementary material for: Visualizing the structure of RNA-seq expression data using grade of membership models
Source: PLoS Genet. 2017 Mar 23;13(3):e1006599. doi: 10.1371/journal.pgen.1006599 (PMC5363805; doi:10.1371/journal.pgen.1006599)

S11 Fig. Sparse Factor Analysis loadings visualization of GTEx brain tissue samples. The colors represent the 6 different factors. The factor loadings are presented in a stacked bar for each sample. We performed SFA under the scenarios of (left) when the loadings are sparse and (right) when the factors are sparse.

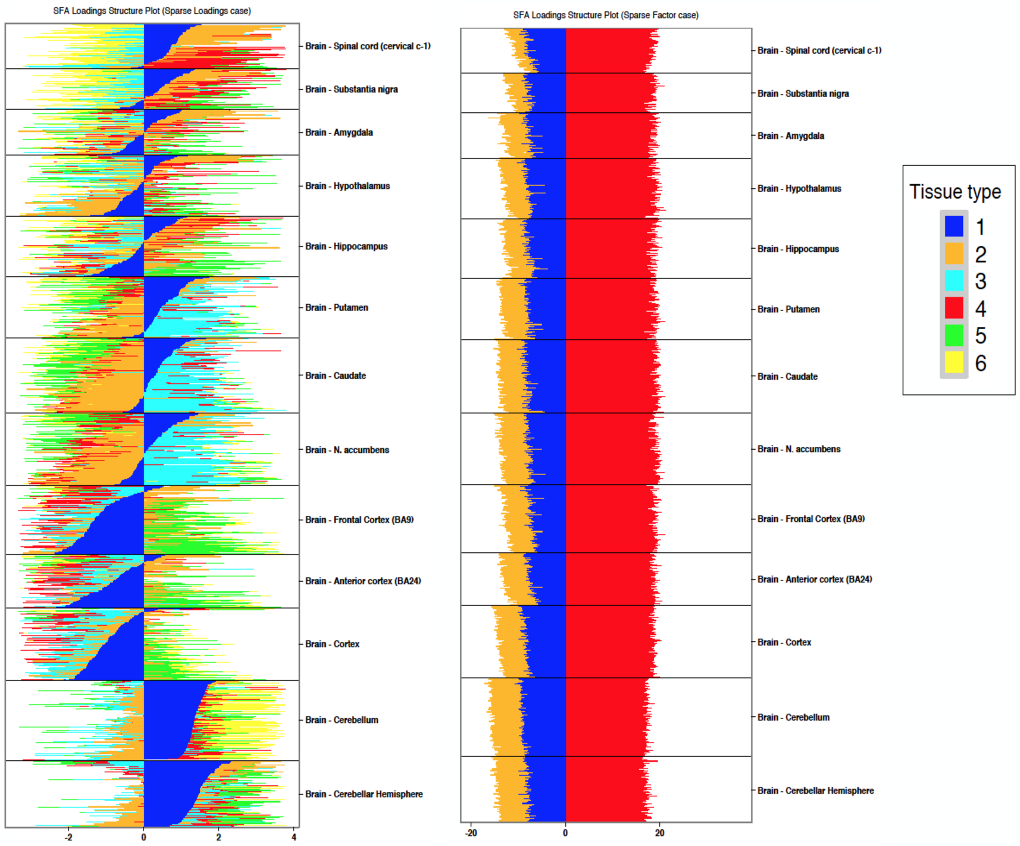

Supplement: S11 Fig — The colors represent the 6 different factors. The factor loadings are presented in a stacked bar for each sample. We performed SFA under the scenarios of when the loadings are sparse (left panel) and when the factors are sparse (right panel). (PDF) [file pgen.1006599.s011.pdf]
