# Supplementary material for: Visualizing the structure of RNA-seq expression data using grade of membership models
Source: PLoS Genet. 2017 Mar 23;13(3):e1006599. doi: 10.1371/journal.pgen.1006599 (PMC5363805; doi:10.1371/journal.pgen.1006599)
Supplement: S1 Table — (PDF) [file pgen.1006599.s013.pdf]

S1 Table. Cluster Annotations of GTEx V6 data with top driving gene summaries.

Cluster	Top Driving Genes	Gene names	Gene Summary
1. Royal purple	*NEAT1*	nuclear paraspeckle assembly transcript 1	produces a long non-coding RNA (lncRNA) transcribed from the multiple endocrine neoplasia locus, regulates genes involved in cancer progression.
	CCNL2	cyclin L2	regulator of the pre-mRNA splicing process, as well as in inducing apoptosis by modulating the expression of apoptotic and antiapoptotic proteins.
	SRSF5	serine/arginine-rich splicing factor 5	encodes proteins of serine/arginine (SR)-rich family, involved in mRNA export from the nucleus and in translation.
2. Light purple	*SNAP25*	synaptosomal-associated protein, 25kDa	this gene product is a presynaptic plasma membrane protein involved in the regulation of neurotransmitter release.
	FBXL16	F-box and leucine-rich repeat protein 16	members of F-box protein family, which interact with SKP1 through the F box, and they interact with ubiquitination targets through other protein interaction domains.
	SLC17A7	neurochondrin	encodes proteins expressed in neuron-rich regions; associated with the membranes of synaptic vesicles and functions in glutamate transport.
3. Red	*FABP4*	fatty acid binding protein 4	encodes the fatty acid binding protein found in adipocytes, takes part in fatty acid uptake, transport, and metabolism.
	PLIN1	perilipin 1	protein encoded by this gene coats lipid storage droplets in adipocytes, thereby protecting them until they can be broken down by hormone-sensitive lipase.
	FASN	fatty acid synthase	catalyze the synthesis of palmitate from acetyl-CoA and malonyl-CoA, in the presence of NADPH, into long-chain saturated fatty acids.
4. Salmon	*ACTG2*	actin, gamma 2, smooth muscle, enteric	involved in various types of cell motility and in the maintenance of the cytoskeleton.
	MYH11	myosin, heavy chain 11, smooth muscle	protein encoded by this gene is a smooth muscle myosin belonging to the myosin heavy chain family, functions as a major contractile protein, converting chemical energy into mechanical energy through the hydrolysis of ATP.
	SYNM	synemin	protein has been found to form a linkage between desmin, which is a subunit of the IF network, and the extracellular matrix, and provides an important structural support in muscle.
5. Denim	*RGS5*	regulator of G-protein signaling 5	encodes a member of the regulators of G protein signaling (RGS) family, associated with retinal arterial macroaneurysm.
	MFGE8	milk fat globule-EGF factor 8 protein	encodes a preproprotein that is proteolytically processed to form multiple protein products, been implicated in wound healing, autoimmune disease, and cancer
	ITGA8	synemin	Proteins generated mediate numerous cellular processes including cell adhesion, cytoskeletal rearrangement, and activation of cell signaling pathways.
6. Light denim	*KRT10*	keratin 10	encodes a member of the type I (acidic) cytokeratin family, mutations associated with epidermolytic hyperkeratosis.
	KRT1	keratin 1, type II	specifically expressed in the spinous and granular layers of the epidermis with family member KRT10 and mutations in these genes have been associated with bullous congenital ichthyosiform erythroderma.
	KRT2	keratin 2, type II	expressed largely in the upper spinous layer of epidermal keratinocytes and mutations in this gene have been associated with bullous congenital ichthyosiform erythroderma.
7. Orange	*NEB*	nebulin	encodes nebulin, a giant protein component of the cytoskeletal matrix that coexists with the thick and thin filaments within the sarcomeres of skeletal muscle, associated with recessive nemaline myopathy.
	MYH1	myosin, heavy chain 1, skeletal muscle, adult	a major contractile protein which converts chemical energy into mechanical energy through the hydrolysis of ATP.
	MYH2	myosin, heavy chain 2, skeletal muscle, adult	encodes a member of the class II or conventional myosin heavy chains, and functions in skeletal muscle contraction.

Cluster	Top Driving Genes	Gene namese	Gene Summary
8. Light orange	*FN1*	fibronectin 1	Fibronectin is involved in cell adhesion, embryogenesis, blood coagulation, host defense, and metastasis.
	COL1A1	collagen, type I, alpha 1	Mutations in this gene associated with osteogenesis imperfecta types I-IV, Ehlers-Danlos syndrome type and Classical type, Caffey Disease.
	COL1A2	collagen, type I, alpha 2	Mutations in this gene associated with osteogenesis imperfecta types I-IV, Ehlers-Danlos syndrome type and Classical type, Caffey Disease.
9. Green	*MBP*	myelin basic protein	major constituent of the myelin sheath of oligodendrocytes and Schwann cells in the nervous system
	GFAP	glial fibrillary acidic protein	encodes one of the major intermediate filament proteins of mature astrocytes, mutations casuses Alexander disease.
	CARNS1	carnosine synthase 1	catalyzes the formation of carnosine and homocarnosine, which are found mainly in skeletal muscle and the central nervous system, respectively.
10. Light green	*CYP17A1*	cytochrome P450 family 17 subfamily A member 1	encodes a member of the cytochrome P450 superfamily of enzymes, mutations in this gene are associated with isolated steroid-17 alpha-hydroxylase deficiency,20-lyase deficiency, pseudohermaphroditism, and adrenal hyperplasia.
	CYP11B1	cytochrome P450 family 11 subfamily B member 1	The protein encoded by this gene plays a key role in the acute regulation of steroid hormone synthesis by enhancing the conversion of cholesterol into pregnenolone, associated with congenital lipoid adrenal hyperplasia.
	GKN1	gastrokine 1	protein encoded by this gene is found to be down-regulated in human gastric cancer tissue as compared to normal gastric mucosa..
11. Turquoise	*MPZ*	myelin protein zero	specifically expressed in Schwann cells of the peripheral nervous system and encodes a type I transmembrane glycoprotein that is a major structural protein of the peripheral myelin sheath, mutations associated with autosomal dominant form of Charcot-Marie-Tooth disease type 1 and other polyneuropathies.
	APOD	apolipoprotein D	encodes a component of high density lipoprotein that has no marked similarity to other apolipoprotein sequences, closely associated with lipoprotein metabolism.
	PMP22	peripheral myelin protein 22	encodes an integral membrane protein that is a major component of myelin in the peripheral nervous system..
12. Yellow	*IGHM*	immunoglobulin heavy constant mu	IgM antibodies play an important role in primary defense mechanisms, Diseases associated with IGHM include agammaglobulinemia 1 and immunodeficiency 23.
	IGHG1	immunoglobulin heavy constant gamma 1 (G1m marker)	antigen binding functionality, diseases associated with IGHG1 include heavy chain deposition disease and chronic lymphocytic leukemia.
	IGHG2	immunoglobulin heavy constant gamma 2 (G2m marker)	antigen binding gene, diseases associated with IGHG2 include c2 deficiency.
13. Sky blue	*TG*	thyroglobulin	thyroglobulin produced predominantly in thyroid gland, synthesizes thyroxine and triiodothyronine, associated with Graves disease and Hashimotot thyroiditis.
	PRL	prolactin 2	encodes the anterior pituitary hormone prolactin. This secreted hormone is a growth regulator for many tissues, including cells of the immune system.
	PRM2	protamine 2	Protamines are the major DNA-binding proteins in the nucleus of sperm.
14. Light pink	*NPPA*	natriuretic peptide A	protein encoded by this gene belongs to the natriuretic peptide family, controls extracellular fluid volume and electrolyte homeostasis, mutations Mutations associated with atrial fibrillation familial type 6.
	MYH6	myosin, heavy chain 6, cardiac muscle, alpha	encodes the alpha heavy chain subunit of cardiac myosin, mutations cause familial hypertrophic cardiomyopathy and atrial septal defect 3
	TNNT2	protamine 2	protein encoded by this gene is the tropomyosin-binding subunit of the troponin complex, mutations in this gene have been associated with familial hypertrophic cardiomyopathy as well as with dilated cardiomyopathy.

Cluster	Top Driving Genes	Gene namese	Gene Summary
15. Light gray	*KRT13*	keratin 13, type I	protein encoded by this gene is a member of the keratin gene family, associated with the autosomal dominant disorder White Sponge Nevus.
	KRT4	keratin 4, type II	protein encoded by this gene is a member of the keratin gene family, associated with White Sponge Nevus, characterized by oral, esophageal, and anal leukoplakia.
	CRNN	cornulin	may play a role in the mucosal/epithelial immune response and epidermal differentiation.
16. Gray	*SFTPB*	surfactant protein B	an amphipathic surfactant protein essential for lung function and homeostasis after birth, muttaions cause pulmonary alveolar proteinosis, fatal respiratory distress in the neonatal period.
	SFTPA2	surfactant protein A2	Mutations in this gene and a highly similar gene located nearby, which affect the highly conserved carbohydrate recognition domain, are associated with idiopathic pulmonary fibrosis.
	SFTPA1	surfactant protein A1	encodes a lung surfactant protein that is a member of C-type lectins called collectins, associated with idiopathic pulmonary fibrosis.
17. Brown	*CSF3R*	colony stimulating factor 3 receptor	protein encoded by this gene is the receptor for colony stimulating factor 3, a cytokine that controls the production, differentiation, and function of granulocytes, mutations a cause of Kostmann syndrome
	MMP25	matrix metallopeptidase 25	proteins are involved in the breakdown of extracellular matrix in normal physiological processes, such as embryonic development, reproduction, and tissue remodeling, as well as in disease processes, such as arthritis and metastasis.
	IL1R2	interleukin 1 receptor type 2	protein encoded by this gene is a cytokine receptor that belongs to the interleukin 1 receptor family.
18. Purple	*PRSS1*	protease, serine 1	secreted by pancreas, associated with pancreatitis
	CPA1	carboxypeptidase A1	secreted by pancreas, linked to pancreatitis and pancreatic cancer
	PNLIP	pancreatic lipase	encodes a carboxyl esterase that hydrolyzes insoluble, emulsified triglycerides, and is essential for the efficient digestion of dietary fats. This gene is expressed specifically in the pancreas.
19. Pink	*HBB*	hemoglobin, beta	mutant beta globin causes sickle cell anemia, absence of beta chain/ reduction in beta globin leads to thalassemia.
	HBA2	hemoglobin, alpha 2	deletion of alpha genes may lead to alpha thalassemia.
	HBA1	hemoglobin, alpha 1	deletion of alpha genes may lead to alpha thalassemia.
20. Dark gray	*ALB*	albumin	functions primarily as a carrier protein for steroids, fatty acids, and thyroid hormones and plays a role in stabilizing extracellular fluid volume.
	HP	haptoglobin	encodes a preproprotein, which subsequently produces haptoglobin, linked to diabetic nephropathy, Crohn's disease, inflammatory disease behavior and reduced incidence of Plasmodium falciparum malaria.
	FGB	fibrinogen beta chain	protein encoded by this gene is the beta component of fibrinogen, mutations may lead to several disorders, including afibrinogenemia, dysfibrinogenemia, hypodysfibrinogenemia etc.
