# Supplementary material for: Visualizing the structure of RNA-seq expression data using grade of membership models
Source: PLoS Genet. 2017 Mar 23;13(3):e1006599. doi: 10.1371/journal.pgen.1006599 (PMC5363805; doi:10.1371/journal.pgen.1006599)
Supplement: S2 Table — (PDF) [file pgen.1006599.s014.pdf]

S2 Table. Cluster Annotations of GTEx V6 Brain data with top driving gene summaries.

Cluster	Top Driving Genes	Gene names	Gene Summary
1, Royal blue	*CLU*	clusterin	protein encoded by this gene is a secreted chaperone that can under some stress conditions also be found in the cell cytosol, also involved in cell death, tumor progression, and neurodegenerative disorders.
	OXT	oxytocin/neurophysin I prepropeptide	encodes a precursor protein that is processed to produce oxytocin and neurophysin I, involved in contraction of smooth muscle during parturition and lactation, cognition, tolerance, adaptation and complex sexual and maternal behaviour.
	GLUL	glutamate-ammonia ligase	catalyzes the synthesis of glutamine from glutamate and ammonia in an ATP-dependent reaction, associated with congenital glutamine deficiency, and overexpression of this gene was observed in some primary liver cancer samples.
2, Turquoise	*ENC1*	ectodermal-neural cortex 1	plays a role in the oxidative stress response as a regulator of the transcription factor Nrf2, may play role in malignant transformation.
	NCALD	neurocalcin delta	encodes a member of the neuronal calcium sensor (NCS), a regulator of G protein-coupled receptor signal transduction.
	YWHAH	tyrosine 3-monooxygenase/tryptophan 5-monooxygenase activation protein eta	mediate signal transduction by binding to phosphoserine-containing proteins, associated with early-onset schizophrenia and psychotic bipolar disorder.
3, Lime green	*PKD1*	polycystin 1, transient receptor potential channel interacting	functions as a regulator of calcium permeable cation channels and intracellular calcium homoeostasis. It is also involved in cell-cell/matrix interactions and may modulate G-protein-coupled signal-transduction pathways.
	CBLN3	cerebellin 3 precursor	contain a cerebellin motif and C-terminal C1q signature domain that mediates trimeric assembly of atypical collagen complexes
	CHGB	chromogranin B	encodes a tyrosine-sulfated secretory protein abundant in peptidergic endocrine cells and neurons. This protein may serve as a precursor for regulatory peptides.
4, Red	*PPP1R1B*	protein phosphatase 1 regulatory inhibitor sub- unit 1B	encodes a bifunctional signal transduction molecule, may serve as a therapeutic target for neurologic and psychiatric disorders.
	RGS14	regulator of G-protein signaling 14	attenuates the signaling activity of G-proteins, increases the rate of conversion of the GTP to GDP.
	NCDN	neurochondrin	encodes a leucine-rich cytoplasmic protein, essential for spatial learning processes.
5, Yellow orange	*MBP*	myelin basic protein	protein encoded is a major constituent of the myelin sheath of oligodendrocytes and Schwann cells in the nervous system.
	GFAP	glial fibrillary acidic protein	encodes major intermediate filament proteins of mature astrocytes, a marker to distinguish astrocytes during development, mutations in this gene cause Alexander disease, a rare disorder of astrocytes in central nervous system.
	TF	transferrin	transport iron from the intestine, reticuloendothelial system, and liver parenchymal cells to all proliferating cells in the body, involved in the removal of certain organic matter and allergens from serum.
6, Yellow	*IQGAP1*	IQ motif containing GTPase activating protein 1	interacts with components of the cytoskeleton, with cell adhesion molecules, and with several signaling molecules to regulate cell morphology and motility.
	A2M	alpha-2-macroglobulin	inhibits many proteases, including trypsin, thrombin and collagenase. A2M is implicated in Alzheimer disease (AD) due to its ability to mediate the clearance and degradation of A-beta, the major component of beta-amyloid deposits.
	C3	complement component 3	plays a central role in the activation of complement system, associated with atypical hemolytic uremic syndrome and age-related macular degeneration in human patients.
