# Supplementary material for: Visualizing the structure of RNA-seq expression data using grade of membership models
Source: PLoS Genet. 2017 Mar 23;13(3):e1006599. doi: 10.1371/journal.pgen.1006599 (PMC5363805; doi:10.1371/journal.pgen.1006599)
Supplement: S3 Table — (PDF) [file pgen.1006599.s015.pdf]

S3 Table. Cluster Annotations of Deng data with top driving genes.

	GO ID	GO Term	Top Driving Genes
1	GO:0007276	gamete generation	BCL2L10; GDF9; NOBOX; PABPC1L; RGS2; CREB3L4; RNF114; BMP15; PTTG1; TDRD12; WEE2; SPIN1; DAZL
2	GO:0007292	female gamete generation	GDF9; BCL2L10; PABPC1L; BMP15; WEE2; DAZL; NOBOX
3	GO:0048609	multicellular organismal reproductive process	GDF9; NOBOX; PABPC1L; BCL2L10; BMP15; CREB3L4; TGFB2; RNF114; RGS2; PTTG1; TDRD12; WEE2; SPIN1; DAZL
4	GO:0032504	multicellular organism reproduction	GDF9; NOBOX; PABPC1L; BCL2L10; BMP15; CREB3L4; TGFB2; RNF114; RGS2; PTTG1; TDRD12; WEE2; SPIN1; DAZL
5	GO:0019953	sexual reproduction	BCL2L10; GDF9; NOBOX; PABPC1L; RGS2; CREB3L4; RNF114; BMP15; PTTG1; TDRD12; WEE2; SPIN1; DAZL
6	GO:0044702	single organism reproductive process	GDF9; NOBOX; PABPC1L; BCL2L10; BMP15; CREB3L4; TGFB2; CASP8; RNF114; RGS2; PTTG1; TDRD12; WEE2; SPIN1; DAZL
7	GO:0048477	oogenesis	WEE2; GDF9; NOBOX; PABPC1L; DAZL
8	GO:0044703	multi-organism reproductive process	BCL2L10; GDF9; NOBOX; PABPC1L; RGS2; CREB3L4; RNF114; BMP15; PTTG1; TDRD12; WEE2; SPIN1; DAZL
9	GO:0048599	oocyte development	WEE2; GDF9; PABPC1L; DAZL
10	GO:0009994	oocyte differentiation	WEE2; GDF9; PABPC1L; DAZL
11	GO:0051321	meiotic cell cycle	H1FOO; WEE2; TDRD12; SPIN1; PTTG1; DAZL
12	GO:0001556	oocyte maturation	WEE2; PABPC1L; DAZL
13	GO:0006306	DNA methylation	TDRD12; H1FOO; TET3; ZFP57
14	GO:0051302	regulation of cell division	TGFB2; PTTG1; TXNIP; WEE2; CHEK1; DAZL
15	GO:0060255	regulation of macromolecule metabolic process	TGFB2; NOBOX; BPGM; UBE2D3; NFYA; CASP8; BMP15; TXNIP; TDRD12; GDF9; BCL2L10

S3 Table continued. Deng et al (2014) Cluster 2 (magenta) top GO annotations.

	GO ID	GO Term	Top Driving Genes
1	GO:0016604	nuclear body	YTHDC1; RBM8A; CDK12; PSME4; PPP1R8; HIPK1; TOPORS
2	GO:0005814	centriole	SFI1; PLK2; ROCK1; TOPORS
3	GO:0044450	microtubule organizing center part	SFI1; PLK2; ROCK1; TOPORS

S3 Table continued. Deng et al (2014) Cluster 3 (yellow) top GO annotations.

	GO ID	GO Term	Top Driving Genes
1	GO:0044428	nuclear part	MAD2L2; SMARCC1; PPRC1; SLU7; NFYB; TOR1B; MIOS; NR1H3; POLR3K
2	GO:0031981	nuclear lumen	MAD2L2; SMARCC1; PPRC1; SLU7; NFYB; POLR1E; MIOS; POLR3K; XPO1
3	GO:0070013	intracellular organelle lumen	MAD2L2; SMARCC1; PPRC1; SLU7; NFYB; POLR1E; MIOS; POLR3K; XPO1; DNTTIP2; ZBTB10; ZBTB17
4	GO:0043233	organelle lumen	MAD2L2; SMARCC1; PPRC1; SLU7; NFYB; POLR1E; MIOS; POLR3K; XPO1
5	GO:0005730	nucleolus	XPO1; DNTTIP2; ESF1; WDR43; ZDHHC7; HEATR1; POLR1E; DDX24; POLR3K
6	GO:0005634	nucleus	MAD2L2; SMARCC1; PPRC1; SLU7; NFYB; TOR1B; MIOS; NR1H3; EIF5B; POLR3K
7	GO:0044446	intracellular organelle part	MAD2L2; PTDSS2; SMARCC1; KLHL21; TOR1B; PPRC1; SLU7; NFYB; SLC25A36; ECE2
8	GO:0005654	nucleoplasm	MAD2L2; SMARCC1; PPRC1; SLU7; NFYB; POLR1E; MIOS; POLR3K; XPO1; ZBTB10; ZBTB17
9	GO:0003723	RNA binding	PPRC1; EIF5B; XPO1; DNTTIP2; WDR43; DDX10; EIF3C; BCLAF1; EBNA1BP2; RARS
10	GO:0003676	nucleic acid binding	SMARCC1; PPRC1; SLU7; NFYB; POLR1E; EIF5B; POLR3K; XPO1; DNTTIP2
11	GO:0043231	intracellular membrane-bounded organelle	MAD2L2; PTDSS2; SMARCC1; TOR1B; PPRC1; SLU7; NFYB; ESF1; ECE2; LMAN1L
12	GO:0043229	intracellular organelle	MAD2L2; PTDSS2; SMARCC1; KLHL21; TOR1B; PPRC1; ARRDC1; SLU7; NFYB; ESF1; ECE2
13	GO:0005874	microtubule	WDR43; KLHL21; HAUS6; CENPE; TEKT2; RACGAP1; WDR81; BCL2L11; KIF20B
14	GO:0044822	poly(A) RNA binding	WDR43; DNTTIP2; ESF1; NXF1; DDX10; HEATR1; EIF3C
15	GO:0044424	intracellular part	MAD2L2; PTDSS2; SMARCC1; KLHL21; TOR1B; PPRC1; SNAPC4; POLR3K; ARRDC1; SLU7; NFYB; ESF1; WDR43; ECE2; LMAN1L

S3 Table continued. Deng et al (2014) Cluster 4 (green) top GO annotations.

	GO ID	GO Term	Top Driving Genes
1	GO:0005829	cytosol	PARG; UAP1; PSMB10; TCEB1; RPLP0; EIF5; CNBP; RPS3; PSAT1; AACS; PMM1; EXOSC7; EIF3I; SET; BHMT; BHMT2
2	GO:0044444	cytoplasmic part	PARG; UAP1; PSMB10; TCEB1; HSPA8; SERINC1; EIF5; CNBP; RPS3; PSAT1; GPD2; AACS; GPR137B; STIP1; PMM1; EXOSC7; VPREB3; PEX16
3	GO:0055131	C3HC4-type RING finger domain binding	HSPA8; PINK1; DNAJA1
4	GO:1901575	organic substance catabolic process	PSMB10; TCEB1; RPLP0; RPS3; GPD2; PINK1; EXOSC7; ALLC; BHMT; HSP90AB1; RPL13A; ATG7; CUL5; UBXN1; ZMPSTE24
5	GO:0000151	ubiquitin ligase complex	DNAJA1; RNF7; UBE2C; HSPA8; FBXO15; SUGT1; DCAF4; CUL5; FBXL20
6	GO:0072655	protein localization to mitochondrion	TIMM17A; BNIP3L; ARIH2; PEMT; SFN; PINK1; HSP90AA1; TIMM23
7	GO:1901564	organonitrogen compound metabolic process	PSMB10; RPLP0; SERINC1; EIF5; BHMT2; PINK1; EIF3I; ALLC; BHMT; MRPL22; RPL13A; ATG7; NUDT9; VNN1; CTSA; HK1
8	GO:0005737	cytoplasm	PARG; UAP1; PSMB10; TCEB1; HSPA8; SERINC1; EIF5; CNBP; RPS3; PSAT1; GPD2; AACS; GPR137B; STIP1; PMM1; EXOSC7
9	GO:0044265	cellular macromolecule catabolic process	EXOSC7; SUMO2; BNIP3L; ARIH2; PSMB10; TCEB1; RPLP0; UBXN1; HSP90AB1; RPL13A; RPS3; RNF7; PINK1
10	GO:0023026	MHC class II protein complex binding	HSP90AB1; HSP90AA1; HSPA8
11	GO:0051082	unfolded protein binding	DNAJA1; PTGES3; HSPA8; HSP90AB1; HSP90AA1; NPM1
12	GO:0009056	catabolic process	PSMB10; TCEB1; RPLP0; RPS3; GPD2; PINK1; EXOSC7; ALLC; WDR45; HSP90AB1; RPL13A
13	GO:0009057	macromolecule catabolic process	EXOSC7; SUMO2; BNIP3L; ARIH2; PSMB10; TCEB1; RPLP0; AZIN1; UBXN1; HSP90AB1; RPL13A
14	GO:0044248	cellular catabolic process	PSMB10; TCEB1; SUMO2; RPS3; GPD2; PINK1; EXOSC7; ALLC; WDR45; HSP90AB1
15	GO:0006626	protein targeting to mitochondrion	TIMM17A; BNIP3L; ARIH2; PEMT; PINK1; HSP90AA1; TIMM23

S3 Table continued. Deng et al (2014) Cluster 5 (purple) top GO annotations.

	GO ID	GO Term	Top Driving Genes
1	GO:0044710	single-organism metabolic process	PCK2; SAT1; EPHX2; NFATC4; CKB; PRDX6; MSH2; EPHA4; PROS1; PDGFRA; PRDX1; UBE2L6; POGLUT1; FABP5; AKAP12; TDGF1; FBP2; SOX2
2	GO:0006950	response to stress	EPHX2; NFATC4; PRDX6; MSH2; EPHA4; PROS1; PDGFRA; PRDX1; UBE2L6; FABP5; TDGF1; SOX2
3	GO:0065010	extracellular membrane-bounded organelle	PCK2; EPHX2; MFGE8; CKB; PRDX6; PROS1; PRDX1; POGLUT1; FABP5; FBP2; TRAP1; PLOD2; DHRS4
4	GO:0070062	extracellular exosome	PCK2; EPHX2; MFGE8; CKB; PRDX6; PROS1; PRDX1; POGLUT1; FABP5; FBP2; TRAP1; PLOD2; DHRS4; MARCKS; DPP4; PRKCI; RAC2; IDH1
5	GO:0043230	extracellular organelle	PCK2; EPHX2; MFGE8; CKB; PRDX6; PROS1; PRDX1; POGLUT1; FABP5; FBP2; TRAP1; PLOD2; DHRS4; MARCKS; DPP4
6	GO:1903561	extracellular vesicle	PCK2; EPHX2; MFGE8; CKB; PRDX6; PROS1; PRDX1; POGLUT1; FABP5; FBP2; TRAP1; PLOD2; DHRS4; MARCKS; DPP4; PRKCI
7	GO:0042221	response to chemical	EPHX2; NFATC4; MFGE8; PRDX6; EPHA4; PROS1; PDGFRA; PRDX1; UBE2L6; TDGF1; SOX2
8	GO:0031988	membrane-bounded vesicle	PCK2; EPHX2; MFGE8; CKB; PRDX6; PROS1; PRDX1; POGLUT1; FABP5; FBP2; TRAP1; PLOD2; DHRS4; SPARC
9	GO:0031982	vesicle	PCK2; EPHX2; MFGE8; CKB; PRDX6; PROS1; PRDX1; POGLUT1; FABP5; FBP2; TRAP1; PLOD2; DHRS4; SPARC
10	GO:0001525	angiogenesis	SAT1; PDGFRA; BMP4; NFATC4; MFGE8; FN1; MEIS1; SPARC; COL4A2; COL4A1; FGF10; TDGF1
11	GO:0048514	blood vessel morphogenesis	SAT1; PDGFRA; BMP4; NFATC4; MFGE8; FN1; ZFP36L1; MEIS1; SPARC; COL4A2; COL4A1; FGF10; TDGF1
12	GO:0001944	vasculature development	SAT1; PDGFRA; BMP4; NFATC4; MFGE8; FN1; ZFP36L1; MEIS1; PDPN; SPARC; COL4A2; COL4A1; FGF10; TDGF1
13	GO:0006979	response to oxidative stress	TAT; PDGFRA; BMP4; ETV5; TRAP1; PRDX6; IDH1; PARP1; AQP8; PRDX1; CRYGD
14	GO:0009725	response to hormone	PRKCI; GJA1; PDGFRA; BMP4; MFGE8; TAT; PLOD2; SPP1; IDH1
15	GO:0030198	extracellular matrix organization	PDGFRA; BMP4; JAM2; FN1; PLOD2; SPARC; SPP1; COL4A2; COL4A1; SERPINH1; DPP4

S3 Table continued. Deng et al (2014) Cluster 6 (orange) top GO annotations.

	GO ID	GO Term	Top Driving Genes
1	GO:0065010	extracellular membrane-bounded organelle	MYH10; SLC2A3; GM2A; TSPAN8; ACTG1; SDC4; TINAGL1; CRYAB; MSN; FABP3; PDZK1IP1; PRSS8; S100A11; DAB2; KRT8; LCP1; UGP2
2	GO:0070062	extracellular exosome	MYH10; SLC2A3; GM2A; TSPAN8; ACTG1; SDC4; TINAGL1; CRYAB; MSN; FABP3; PDZK1IP1; PRSS8; S100A11; DAB2; KRT8; LCP1; UGP2
3	GO:0043230	extracellular organelle	MYH10; SLC2A3; GM2A; TSPAN8; ACTG1; SDC4; TINAGL1; CRYAB; MSN; FABP3; PDZK1IP1; PRSS8; S100A11
4	GO:1903561	extracellular vesicle	MYH10; SLC2A3; GM2A; TSPAN8; ACTG1; SDC4; TINAGL1; CRYAB; MSN; FABP3; PDZK1IP1; PRSS8; S100A11; DAB2; KRT8
5	GO:0031988	membrane-bounded vesicle	MYH10; SLC2A3; GM2A; TSPAN8; ACTG1; TMSB4X; SDC4; TINAGL1; CRYAB; MSN; FABP3; PDZK1IP1; PRSS8; S100A11; DAB2
6	GO:0031982	vesicle	MYH10; SLC2A3; GM2A; TSPAN8; ACTG1; TMSB4X; SDC4; TINAGL1; CRYAB; MSN; FABP3; PDZK1IP1; PRSS8; S100A11; DAB2; KRT8
7	GO:0008092	cytoskeletal protein binding	MYH10; TPM4; TMSB4X; CRYAB; MSN; TMSB10; FABP3; NDRG1; CALM1; FMNL2; MYH9; CAP1; TPM1; CDH1
8	GO:0015629	actin cytoskeleton	MYH10; CLIC4; MYH9; MYL12B; WDR1; CNN2; ARPC2; AHNAK; ACTN4; CRYAB; CAP1; TPM1; DSTN; ARPC5; TPM4
9	GO:0003779	actin binding	MYH10; TPM4; WDR1; CNN2; FMNL2; ARPC2; MYH9; CAP1; TPM1
10	GO:0048468	cell development	MYH10; CAPG; ACTG1; WDR1; CNN2; FMNL2; MYH9; ACTN4; SDC4; CAP1; TPM1; DSTN
11	GO:0030036	actin cytoskeleton organization	MYH10; CAPG; ACTG1; WDR1; CNN2; FMNL2; MYH9; ACTN4; SDC4; CAP1; TPM1
12	GO:0032432	actin filament bundle	MYH10; TPM4; MYL12B; CNN2; MYH9; CRYAB; TPM1; ACTN4; LCP1
13	GO:0005912	adherens junction	TJP2; MYH9; ACTG1; CNN2; ARPC2; AHNAK; ACTN4; SDC4
14	GO:0070161	anchoring junction	TJP2; MYH9; ACTG1; CNN2; ARPC2; AHNAK; ACTN4; SDC4
15	GO:0005925	focal adhesion	MYH9; ACTG1; CNN2; ARPC2; AHNAK; ACTN4; SDC4; CAP1; ARPC5
