# Supplementary material for: Visualizing the structure of RNA-seq expression data using grade of membership models
Source: PLoS Genet. 2017 Mar 23;13(3):e1006599. doi: 10.1371/journal.pgen.1006599 (PMC5363805; doi:10.1371/journal.pgen.1006599)
Supplement: S4 Table — (PDF) [file pgen.1006599.s016.pdf]

**S4 Table. Cluster Annotation of Deng data analysis using 48 genes with top driving gene summaries.**

| Cluster | Top 5 Driving Genes | Top significant GO terms (function)[q-value] |
|---|---|---|
| Green | *Actb* | GO:0048568 (embryonic organ development)[9e-08], GO:0048468 (cell development)[4e-07], GO:0001890 (placenta development)[1e-06], GO:0051094 (positive regulation of developmental process)[1e-06], GO:0030097 (hemopoiesis)[1e-05] |
| Purple | *Pecam1, Esrrb, Fn1, Pdgfra, Sox2* | GO:0048864 (stem cell development)[4e-12], GO:0048863 (stem cell differentiation)[2e-11], GO:0009893 (positive regulation of metabolic process)[7e-10], GO:0009653 ( anatomical structure morphogenesis)[4e-08] |
| Orange | *Dppa1, Gata3, Id2, Dab2, Lcp1* | GO:0061061 (muscle structure development)[2e-13], GO:0060537 (muscle tissue development)[2e-12], GO:0048514 (blood vessel morphogenesis)[8e-12], GO:0007275 (multocellular orgasnimal development) |
