# Supplementary material for: Correction: Visualizing the structure of RNA-seq expression data using grade of membership models
Source: PLoS Genet. 2017 May 26;13(5):e1006759. doi: 10.1371/journal.pgen.1006759 (PMC5446108; doi:10.1371/journal.pgen.1006759)
Supplement: S1 File — (PDF) [file pgen.1006759.s001.pdf]

PLOS | GENETICS

RESEARCH ARTICLE

Visualizing the structure of RNA-seq expression data using grade of membership models

Kushal K. Dey[1], **Chiaowen Joyce Hsiao**[2], **Matthew Stephens**[1,2]*

1 Department of Statistics, University of Chicago, Chicago, Illinois, United States of America, **2** Department of Human Genetics, University of Chicago, Chicago, Illinois, United States of America

* mstephens@uchicago.edu

Check for updates

OPEN ACCESS

Citation: Dey KK, Hsiao CJ, Stephens M (2017) Visualizing the structure of RNA-seq expression data using grade of membership models. PLoS Genet 13(3): e1006599. doi:10.1371/journal.pgen.1006599

Editor: Anshul Kundaje, Stanford University, UNITED STATES

Received: May 30, 2016

Accepted: January 24, 2017

Published: March 23, 2017

Copyright: © 2017 Dey et al. This is an open access article distributed under the terms of the Creative Commons Attribution License, which permits unrestricted use, distribution, and reproduction in any medium, provided the original author and source are credited.

Data Availability Statement: Links to the data used in the analysis, along with references, are provided in the main text of the manuscript.

Funding: The Genotype-Tissue Expression (GTEx) Project was supported by the Common Fund of the Office of the Director of the National Institutes of Health. Additional funds were provided by the NCI, NHGRI, NHLBI, NIDA, NIMH, and NINDS. Donors were enrolled at Biospecimen Source Sites funded by NCI SAIC-Frederick, Inc. (SAIC-F) subcontracts to the National Disease Research Interchange (10XS170), Roswell Park Cancer Institute

Abstract

Grade of membership models, also known as "admixture models", "topic models" or "Latent Dirichlet Allocation", are a generalization of cluster models that allow each sample to have membership in multiple clusters. These models are widely used in population genetics to model admixed individuals who have ancestry from multiple "populations", and in natural language processing to model documents having words from multiple "topics". Here we illustrate the potential for these models to cluster samples of RNA-seq gene expression data, measured on either bulk samples or single cells. We also provide methods to help interpret the clusters, by identifying genes that are distinctively expressed in each cluster. By applying these methods to several example RNA-seq applications we demonstrate their utility in identifying and summarizing structure and heterogeneity. Applied to data from the GTEx project on 53 human tissues, the approach highlights similarities among biologically-related tissues and identifies distinctively-expressed genes that recapitulate known biology. Applied to single-cell expression data from mouse preimplantation embryos, the approach highlights both discrete and continuous variation through early embryonic development stages, and highlights genes involved in a variety of relevant processes—from germ cell development, through compaction and morula formation, to the formation of inner cell mass and trophoblast at the blastocyst stage. The methods are implemented in the Bioconductor package `CountClust`.

Author summary

Gene expression profile of a biological sample (either from single cells or pooled cells) results from a complex interplay of multiple related biological processes. Consequently, for example, distal tissue samples may share a similar gene expression profile through some common underlying biological processes. Our goal here is to illustrate that grade of membership (GoM) models—an approach widely used in population genetics to cluster admixed individuals who have ancestry from multiple populations—provide an attractive approach for clustering biological samples of RNA sequencing data. The GoM model allows each biological sample to have partial memberships in multiple biologically-distinct

(10XS171), and Science Care, Inc. (X10S172). The Laboratory, Data Analysis, and Coordinating Center (LDACC) was funded through a contract (HHSN268201000029C) to The Broad Institute, Inc. Biorepository operations were funded through an SAIC-F subcontract to Van Andel Institute (10ST1035). Additional data repository and project management were provided by SAIC-F (HHSN261200800001E). The Brain Bank was supported by a supplements to University of Miami grants DA006227 & DA033684 and to contract N01MH000028. Statistical Methods development grants were made to the University of Geneva (MH090941 & MH101814), the University of Chicago (MH090951, MH090937, MH101820, MH101825), the University of North Carolina - Chapel Hill (MH090936 & MH101819), Harvard University (MH090948), Stanford University (MH101782), Washington University St Louis (MH101810), and the University of Pennsylvania (MH101822). The paper is supported by the grant U01CA198933 from the NIH BD2K program. The funders had no role in study design, data collection and analysis, decision to publish, or preparation of the manuscript.

Competing interests: The authors have declared that no competing interests exist.

clusters, in contrast to traditional clustering methods that partition samples into distinct subgroups. We also provide methods for identifying genes that are distinctively expressed in each cluster to help biologically interpret the results. Applied to a dataset of 53 human tissues, the GoM approach highlights similarities among biologically-related tissues and identifies distinctively-expressed genes that recapitulate known biology. Applied to gene expression data of single cells from mouse preimplantation embryos, the approach highlights both discrete and continuous variation through early embryonic development stages, and genes involved in a variety of relevant processes. Our study highlights the potential of GoM models for elucidating biological structure in RNA-seq gene expression data.

Introduction

Ever since large-scale gene expression measurements have been possible, clustering—of both genes and samples—has played a major role in their analysis [1–3]. For example, clustering of genes can identify genes that are working together or are co-regulated, and clustering of samples is useful for quality control as well as identifying biologically-distinct subgroups. A wide range of clustering methods have therefore been employed in this context, including distance-based hierarchical clustering, *k*-means clustering, and self-organizing maps (SOMs); see for example [4, 5] for reviews.

Here we focus on cluster analysis of samples, rather than clustering of genes (although our methods do highlight sets of genes that distinguish each cluster). Traditional clustering methods for this problem attempt to partition samples into distinct groups that show "similar" expression patterns. While partitioning samples in this way has intuitive appeal, it seems likely that the structure of a typical gene expression data set will be too complex to be fully captured by such a partitioning. Motivated by this, here we analyse expression data using grade of membership (GoM) models [6], which generalize clustering models to allow each sample to have partial membership in multiple clusters. That is, they allow that each sample has a proportion, or "grade" of membership in each cluster. Such models are widely used in population genetics to model admixture, where individuals can have ancestry from multiple populations [7], and in document clustering [8, 9] where each document can have membership in multiple topics. In these fields GoM models are often known as "admixture models", and "topic models" or "Latent Dirichlet Allocation" [8]. GoM models have also recently been applied to detect mutation signatures in cancer samples [10].

Although we are not the first to apply GoM-like models to gene expression data, previous applications have been primarily motivated by a specific goal, "cell type deconvolution", which involves using cell-type-specific expression profiles of marker genes to estimate the proportions of different cell types in a mixture [11–13]. Specifically, the GoM model we use here is analogous to—although different in detail from—blind deconvolution approaches [14–16] which estimate cell type proportions and cell type signatures jointly (see also [17, 18] for semi-supervised approaches). Our goal here is to demonstrate that GoM models can be useful much more broadly for understanding structure in RNA-seq data—not only to deconvolve mixtures of cell types. For example, in our analysis of human tissue samples from the GTEX project below, the GoM model usefully captures biological heterogeneity among samples even though the inferred grades of membership are unlikely to correspond precisely to proportions of specific cell types. And in our analyses of single-cell expression data the GoM model highlights interesting structure, even though interpreting the grades of membership

as "proportions of cell types" is clearly inappropriate because each sample is a single cell! Here we are exploiting the GoM as a flexible extension of traditional cluster models, which can capture "continuous" variation among cells as well as the more "discrete" variation captured by cluster models. Indeed, the extent to which variation among cells can be described in terms of discrete clusters versus more continuous populations is a fundamental question that, when combined with appropriate single-cell RNA-seq data, the GoM models used here may ultimately help address.

Methods overview

We assume that the RNA-seq data on N samples has been summarized by a table of counts $C_{N\times G} = (c_{ng})$, where c_{ng} is the number of reads from sample n mapped to gene g (or other unit, such as transcript or exon) [19]. The GoM model is a generalization of a cluster model, which allows that each sample has some proportion ("grade") of membership, in each cluster. For RNA-seq data this corresponds to assuming that each sample n has some proportion of its reads, q_{nk} coming from cluster k. In addition, each cluster k is characterized by a probability vector, $\theta_{k\cdot}$, whose gth element represents the relative expression of gene g in cluster k. The GoM model is then

$$(c_{n1}, c_{n2}, \cdots, c_{nG}) \sim \text{Multinomial}\big(c_{n+}, p_{n1}, p_{n2}, \cdots, p_{nG}\big), \tag{1}$$

where

$$p_{ng} := \sum_{k=1}^{K} q_{nk}\theta_{kg}. \tag{2}$$

The number of clusters K is set by the analyst, and it can be helpful to explore multiple values of K (see Discussion).

To fit this model to RNA-seq data, we exploit the fact that this GoM model is commonly used for document clustering [8]. This is because, just as RNA-seq samples can be summarized by counts of reads mapping to each possible gene in the genome, document data can be summarized by counts of each possible word in a dictionary. Recognizing this allows existing methods and software for document clustering to be applied directly to RNA-seq data. Here we use the R package `maptpx` [20] to fit the GoM model.

Fitting the GoM model results in estimated membership proportions q for each sample, and estimated expression values θ for each cluster. We visualize the membership proportions for each sample using a "Structure plot" [21], which is named for its widespread use in visualizing the results of the *Structure* software [7] in population genetics. The Structure plot represents the estimated membership proportions of each sample as a stacked barchart, with bars of different colors representing different clusters. Consequently, samples that have similar membership proportions have similar amounts of each color. See Fig 1 for example.

To help biologically interpret the clusters inferred by the GoM model we also implemented methods to identify, for each cluster, which genes are most distinctively differentially expressed in that cluster; that is, which genes show the biggest difference in expression compared with the other most similar cluster (see Methods). Functions for fitting the GoM model, plotting the structure plots, and identifying the distinctive ("driving") genes in each cluster, are included in our R package `CountClust` [22] available through Bioconductor [23].

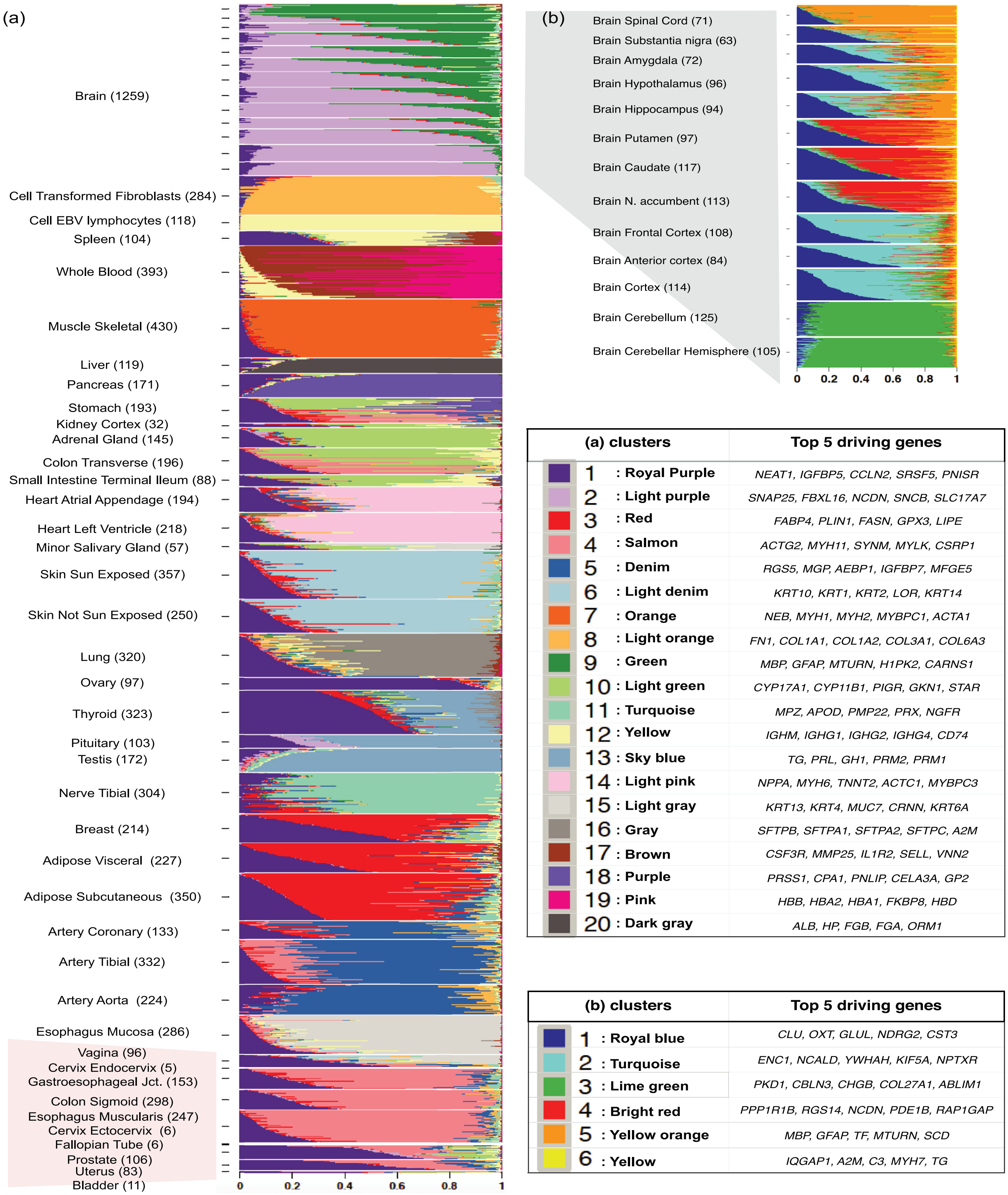

(a) clusters	Top 5 driving genes
1 : Royal Purple	*NEAT1, IGFBP5, CCLN2, SRSF5, PNISR*
2 : Light purple	*SNAP25, FBXL16, NCDN, SNCB, SLC17A7*
3 : Red	*FABP4, PLIN1, FASN, GPX3, LIPE*
4 : Salmon	*ACTG2, MYH11, SYNM, MYLK, CSRP1*
5 : Denim	*RGS5, MGP, AEBP1, IGFBP7, MFGE5*
6 : Light denim	*KRT10, KRT1, KRT2, LOR, KRT14*
7 : Orange	*NEB, MYH1, MYH2, MYBPC1, ACTA1*
8 : Light orange	*FN1, COL1A1, COL1A2, COL3A1, COL6A3*
9 : Green	*MBP, GFAP, MTURN, H1PK2, CARNS1*
10 : Light green	*CYP17A1, CYP11B1, PIGR, GKN1, STAR*
11 : Turquoise	*MPZ, APOD, PMP22, PRX, NGFR*
12 : Yellow	*IGHM, IGHG1, IGHG2, IGHG4, CD74*
13 : Sky blue	*TG, PRL, GH1, PRM2, PRM1*
14 : Light pink	*NPPA, MYH6, TNNT2, ACTC1, MYBPC3*
15 : Light gray	*KRT13, KRT4, MUC7, CRNN, KRT6A*
16 : Gray	*SFTPB, SFTPA1, SFTPA2, SFTPC, A2M*
17 : Brown	*CSF3R, MMP25, IL1R2, SELL, VNN2*
18 : Purple	*PRSS1, CPA1, PNLIP, CELA3A, GP2*
19 : Pink	*HBB, HBA2, HBA1, FKBP8, HBD*
20 : Dark gray	*ALB, HP, FGB, FGA, ORM1*

(b) clusters	Top 5 driving genes
1 : Royal blue	*CLU, OXT, GLUL, NDRG2, CST3*
2 : Turquoise	*ENC1, NCALD, YWHAH, KIF5A, NPTXR*
3 : Lime green	*PKD1, CBLN3, CHGB, COL27A1, ABLIM1*
4 : Bright red	*PPP1R1B, RGS14, NCDN, PDE1B, RAP1GAP*
5 : Yellow orange	*MBP, GFAP, TF, MTURN, SCD*
6 : Yellow	*IQGAP1, A2M, C3, MYH7, TG*

Fig 1. GTEx tissues Structure plot. (a): Structure plot of estimated membership proportions for GoM model with K = 20 clusters fit to 8, 555 tissue samples from 53 tissues in GTEx data. Each horizontal bar shows the cluster membership proportions for a single sample, ordered so that samples from the same tissue are adjacent to one another. Within each tissue, the samples are sorted by the proportional representation of the underlying

clusters. (b): Structure plot of estimated membership proportions for K = 4 clusters fit to only the brain tissue samples. This analysis highlights finer-scale structure among the brain samples that is missed by the global analysis in (a).

doi:10.1371/journal.pgen.1006599.g001

Results

Bulk RNA-seq data of human tissue samples

We begin by illustrating the GoM model on bulk RNA expression measurements from the GTEx project (V6 dbGaP accession phs000424.v6.p1, release date: Oct 19, 2015, http://www.gtexportal.org/home/). These data consist of per-gene read counts from RNA-seq performed on 8,555 samples collected from 450 human donors across 53 tissues, lymphoblastoid cell lines, and transformed fibroblast cell-lines. We analyzed 16,069 genes that satisfied filters (e.g. exceeding certain minimum expression levels) that were used during eQTL analyses by the GTEx project (gene list available in http://stephenslab.github.io/count-clustering/project/utilities/gene_names_all_gtex.txt).

We fit the GoM model to these data, with number of clusters $K = 5, 10, 15, 20$. For each K we ran the fitting algorithm three times and kept the result with the highest log-likelihood. As might be expected, increasing K highlights finer structure in the data, and for brevity we focus discussion on results for $K = 20$ (Fig 1(a)), with results for other K shown in S1 Fig. For comparison we also ran several other commonly-used methods for clustering and visualizing gene expression data: Principal Components Analysis (PCA), Multidimensional Scaling (MDS), *t*-Distributed Stochastic Neighbor Embedding (*t*-SNE) [24, 25], and hierarchical clustering (Fig 2).

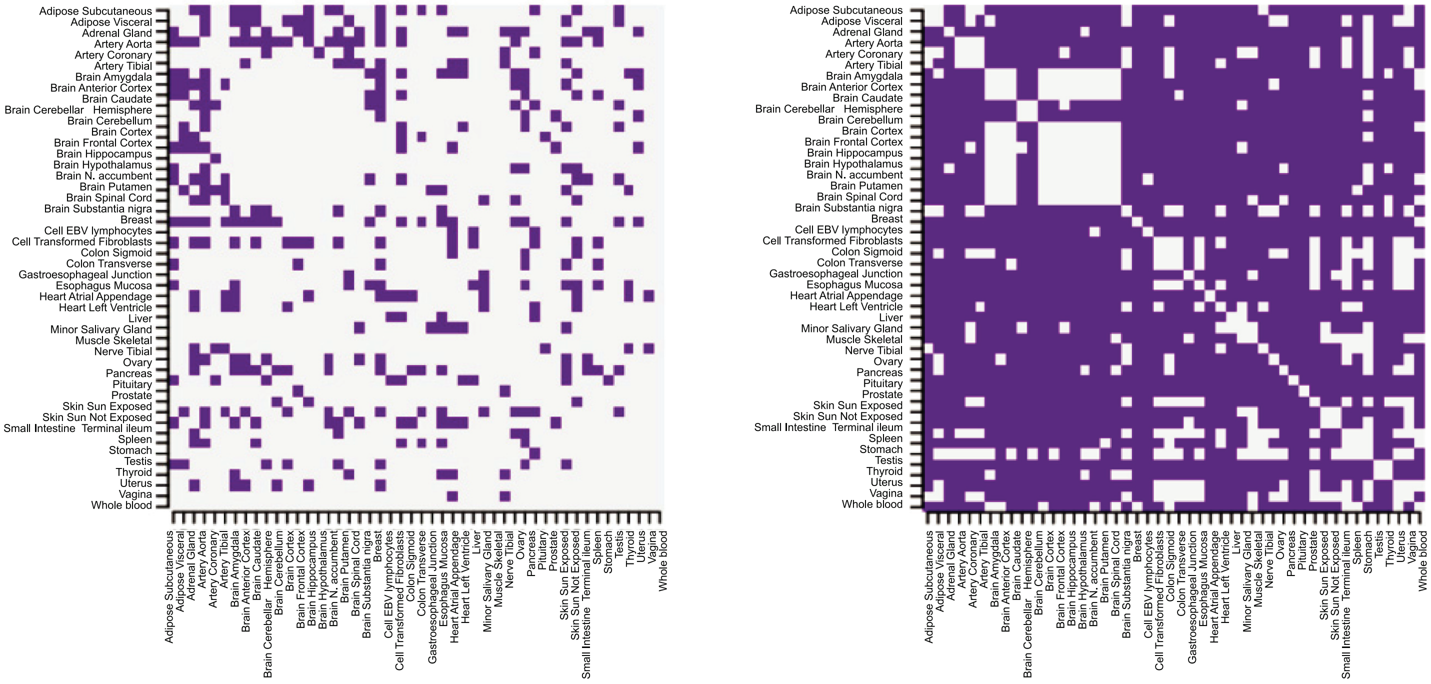

(a) hierarchy method

(b) GoM method

Fig 2. A comparison of accuracy of GoM model vs hierarchical clustering. For each pair of tissues from the GTEX data we assessed whether or not each method (with K = 2 clusters) separated the samples precisely according to their actual tissue of origin, with successful separation indicated by a filled square. Some pairs of tissues (e.g. pairs of brain tissues) are more difficult to distinguish than others. Overall the GoM model is successful in 86.

doi:10.1371/journal.pgen.1006599.g002

These data present a challenge to visualization and clustering tools, because of both the relatively large number of samples and the complex structure created by the inclusion of many different tissues. Indeed, neither PCA nor MDS provide satisfactory summaries of the structure in these data (Fig 2(a) and 2(b)): samples from quite different tissues are often super-imposed on one another in plots of PC1 vs PC2, and this issue is only partly alleviated by examining more PCs (S2 Fig). The hierarchical clustering provides perhaps better separation of tissues (Fig 2(d)), but producing a clear (static) visualization of the tree is difficult with this many samples. By comparison *t*-SNE (Fig 2(b)) and the GoM model (Fig 1(a)) both show a much clearer visual separation of samples by tissue, although they achieve this in very different ways. The *t*-SNE representation produces a two-dimensional plot with 20–25 visually-distinct clusters. In contrast, the GoM highlights similarity among samples by assigning them similar membership proportions, resulting in groups of similarly-colored bars in the structure plot. Some tissues are represented by essentially a single cluster/color (e.g. Pancreas, Liver), whereas other tissues are represented as a mixture of multiple clusters (e.g. Thyroid, Spleen). Furthermore, the GoM results highlight biological similarity among some tissues by assigning similar membership proportions to samples from those tissues. For example, samples from several different parts of the brain often have similar memberships, as do the arteries (aorta, tibial and coronary) and skin samples (sun-exposed and un-exposed).

Although it is not surprising that samples cluster by tissue, other results could have occurred. For example, samples could have clustered according to technical variables, such as sequencing batch [26] or sample collection center. While our results do not exclude the possibility that technical variables could have influenced these data, the *t*-SNE and GoM results clearly demonstrate that tissue of origin is the primary source of heterogeneity, and provide a useful initial assurance of data quality.

While in these data both the GoM model and *t*-SNE highlight the primary structure due to tissue of origin, the GoM results have at least two advantages over *t*-SNE. First, the GoM model provides an explicit, quantitative, estimate of the mean expression of each gene in each cluster, making it straightforward to assess which genes and processes drive differences among clusters; see Table 1 (and also S1 Table). Reassuringly, many results align with known biology. For example, the purple cluster (cluster 18), which distinguishes Pancreas from other tissues, is enriched for genes responsible for digestion and proteolysis, (e.g. *PRSS1*, *CPA1*, *PNLIP*). Similarly the yellow cluster (cluster 12), which primarily distinguishes Cell EBV Lymphocytes from other tissues, is enriched with genes responsible for immune responses (e.g. *IGHM*, *IGHG1*) and the pink cluster (cluster 19) which mainly appears in Whole Blood, is enriched with genes related hemoglobin complex and oxygen transport (e.g. *HBB*, *HBA1*, *HBA2*). Further, Keratin-related genes characterize the skin cluster (cluster 6, light denim), Myosin-related genes characterize the muscle skeletal cluster (cluster 7, orange), etc. These biological annotations are particularly helpful for understanding instances where a cluster appears in multiple tissues. For example, the top genes in the salmon cluster (cluster 4), which is common to the Gastroesophageal Junction, Esophagus Muscularis and Colon Sigmoid, are related to smooth muscle. And the top genes in the red cluster, highlighted above as common to Breast Mammary tissue, Adipose Subcutaneous and Adipose Visceral, are all related to adipocytes and/or fatty acid synthesis.

A second advantage of the GoM model is that, because it allows partial membership in each cluster, it is better able to highlight partial similarities among distinct tissues. For example, in Fig 1(a) the sky blue cluster (cluster 13), appears in testis, pituitary, and thyroid, reflecting shared hormonal-related processes. At the same time, these tissues are distinguished from one another both by their degree of membership in this cluster (testis samples have consistently stronger membership; thyroid samples consistently weaker), and by membership in other

Table 1. Cluster Annotations GTEx V6 data (with GO annotations).

Cluster	Top 5 Driving Genes	Top significant GO terms (function)[q-value]
1. Royal purple	*NEAT1, IGFBP5, CCLN2, SRSF5, PNISR*	GO:0005654 (nucleoplasm)[2e-10], GO:0044822 (poly-A RNA binding)[3e-09], GO:0044428 (nuclear part)[1e-09], GO:0043233 (organelle lumen)[2e-08]
2. Light purple	*SNAP25, FBXL16, NCDN, SNCB, SLC17A7*	GO:0097458 (neuron part)[2e-25], GO:0007268 (synaptic transmission)[9e-18], GO:0030182 (neuron differentiation)[2e-14], GO:0022008 (neurogenesis)[1e-13], GO:0007267 (cell-cell signaling)[3e-13]
3. Red	*FABP4, PLIN1, FASN, GPX3, LIPE*	GO:0044255 (cellular lipid metabolism)[1e-09], GO:0006629 (lipid metabolism)[1e-09], GO:0006639 (acylglycerol metabolism)[3e-08], GO:0045765 (angiogenesis regulation)[4e-08]
4. Salmon	*ACTG2, MYH11, SYNM, MYLK, CSRP1*	GO:0043292 (contractile fiber)[3e-13], GO:0006936 (muscle contraction)[5e-12], GO:0030016 (myofibril)[5e-12], GO:0015629 (actin cytoskeleton)[2e-12], GO:0005925 (focal adhesion)[6e-11]
5. Denim	*RGS5, MGP, AEBP1, IGFBP7, MFGE8*	GO:0005578 (proteinaceous extracellular matrix)[4e-20], GO:0030198 (extracellular matrix)[2e-18], GO:0007155 (cell adhesion)[4e-14], GO:0001568 (blood vessel development)[4e-13]
6. Light denim	*KRT10, KRT1, KRT2, LOR, KRT14*	GO:0008544 (epidermis development)[3e-12], GO:0043588 (skin development)[5e-10], GO:0042303 (molting cycle)[8e-06], GO:0042633 (hair cycle)[7e-06], GO:0048513 (organ development)[6e-05]
7. Orange	*NEB, MYH1, MYH2, MYBPC1, ACTA1*	GO:0043292 (contractile fiber)[6e-52], GO:0030016 (myofibril)[1e-51], GO:0030017 (sarcomere)[5e-40], GO:0003012 (muscle system process)[2e-25], GO:0015629 (actin cytoskeleton)[1e-25]
8. Light orange	*FN1, COL1A1, COL1A2, COL3A1, COL6A3*	GO:0030198 (extracellular matrix)[6e-29], GO:0043062 (extracellular structure)[4e-29], GO:0032963 (collagen metabolism)[3e-16], GO:0030199 (collagen fibril organization)[1e-14], GO:0030574 (collagen catabolism)[1e-14]
9. Green	*MBP, GFAP, MTURN, HIPK2, CARNS1*	GO:0043209 (myelin sheath)[4e-07], GO:0007399 (nervous system development)[4e-05], GO:0008366 (axon ensheathment)[9e-05], GO:0044430 (cytoskeletal part)[1e-04], GO:0005874 (microtubule)[3e-04]
10. Light green	*CYP17A1, CYP11B1, PIGR, GKN1, STAR*	GO:0006694 (steroid biosynthesis)[2e-13], GO:0008202 (steroid metabolism)[1e-12], GO:0016125 (sterol metabolism)[1e-11], GO:0042446 (hormone biosynthesis)[1e-10], GO:0008207 (C21-steroid hormone metabolism)[3e-10]
11. Turquoise	*MPZ, APOD, PMP22, PRX, NGFR*	GO:0007272 (ensheathment of neurons)[4e-07], GO:0008366 (axon ensheathment)[7e-07], GO:0042552 (myelination)[7e-06], GO:0048856 (anatomical structure development)[1e-06], GO:0005578 (proteinaceous extracellular matrix)[1e-06]
12. Yellow	*IGHM, IGHG1, IGHG2, IGHG4, CD74*	GO:0006955 (immune response)[1e-18], GO:0002252 (immune effector process)[7e-18], GO:0003823 (antigen binding)[1e-15], GO:0019724 (B-cell mediated immunity)[5e-13], GO:0002684 (positive regulation immune system)[6e-13]
13. Sky blue	*TG, PRL, GH1, PRM2, PRM1*	GO:0019953 (sexual reproduction)[8e-10], GO:0048232 (male gamete generation)[2e-08], GO:0035686 (sperm fibrous sheath)[4e-06], GO:0005179 (hormone activity)[6e-05], GO:0042403 (thyroid hormone metabolism)[2e-04]
14. Light pink	*NPPA, MYH6, TNNT2, ACTC1, MYBPC3*	GO:0045333 (cellular respiration)[2e-34], GO:0022904 (respiratory electron transport)[8e-33], GO:0015980 (energy derivation by oxidation of organic compounds)[4e-30], GO:0031966 (mitochondrial membrane)[5e-26]
15. Light gray	*KRT13, KRT4, MUC7, CRNN, KRT6A*	GO:0070062 (extracellular exosome)[2e-23], GO:0043230 (extracellular organelle)[3e-23], GO:0031982 (vesicle)[3e-20], GO:0008544 (epidermis development)[2e-18], GO:0043588 (skin development)[1e-13]
16. Gray	*SFTPBβ, SFTPA1, SFTPA2, SFTPC, A2M*	GO:0001525 (angiogenesis)[5e-08], GO:0001944 (vasculature development)[2e-07], GO:0048514 (blood vessel morphogenesis)[2e-07], GO:0040012 (locomotion regulation)[4e-06], GO:2000145 (cell motility)[1e-05]
17. Brown	*CSF3R, MMP25, IL1R2, SELL, VNN2*	GO:0006955 (immune response)[8e-22], GO:0006952 (defense response)[9e-16], GO:0071944 (cell periphery)[7e-15], GO:0005886 (plasma membrane)[7e-15], GO:0050776 (regulation of immune response)[2e-12]
18. Purple	*PRSS1, CPA1, PNLIP, CELA3A, GP2*	GO:0007586 (digestion)[3e-14], GO:0004252 (serine-type endopeptidase activity)[4e-08], GO:0006508 (proteolysis)[6e-06], GO:0016787 (hydrolase activity)[6e-05], GO:0044241 (lipid digestion)[1e-04]
19. Pink	*HBB, HBA2, HBA1, FKBP8, HBD*	GO:0005833 (hemoglobin complex)[1e-13], GO:0015669 (gas transport)[4e-11], GO:0020037 (heme binding)[3e-07], GO:0031720 (haptoglobin binding)[3e-06], GO:0006950 (response to stress)[6e-04]
20. Dark gray	*ALB, HP, FGB, FGA, ORM1*	GO:0072562 (blood microparticle)[1e-27], GO:0043230 (extracellular organelle)[1e-24], GO:0044710 (single organism metabolism)[7e-20], GO:0019752 (carboxylic acid metabolism)[1e-18], GO:0034364 (high density lipoprotein)[3e-16]

doi:10.1371/journal.pgen.1006599.t001

clusters. For example, pituitary samples, but not testis or thyroid samples, have membership in the light purple cluster (cluster 2) which is driven by genes related to neurons and synapsis. In the *t*-SNE results these three tissues simply cluster separately into visually distinct groups, with no indication that their expression profiles have something in common (Fig 2(b)). Thus, although we find the *t*-SNE results visually attractive, this 2-dimensional projection contains less information than the Structure plot from the GoM (Fig 1(a)), which uses color to represent the samples in a 20-dimensional space.

In addition to these qualitative comparisons with other methods, we also used the GTEx data to quantitatively compare the accuracy of the GoM model with hierarchical clustering. Specifically, for each pair of tissues in the GTEx data we assessed whether or not each method correctly partitioned samples into the two tissue groups; see Methods. (Other methods do not provide an explicit clustering of the samples—only a visual representation—and so are not included in these comparisons.) The GoM model was more accurate in this test, succeeding in 88% of comparisons, compared with 79% for hierarchical clustering (S3(a) vs S3(c) Fig).

Sub-analysis of brain tissues

Although the analysis of all tissues is useful for assessing global structure, it may miss finer-scale structure within tissues or among similar tissues. For example, here the GoM model applied to all tissues effectively allocated only three clusters to all brain tissues (clusters 1,2 and 9 in Fig 1(a)), and we suspected that additional substructure might be uncovered by analyzing the brain samples separately and using more clusters. Fig 1(b) shows the Structure plot for $K = 6$ on only the Brain samples. The results highlight much finer-scale structure compared with the global analysis; see Table 2. Brain Cerebellum and Cerebellar hemisphere are essentially assigned to a separate cluster (lime green), which is enriched with genes related to cell periphery and communication (e.g. *PKD1*, *CBLN3*) as well as genes expressed largely in neuronal cells and playing a role in neuron differentiation (e.g. *CHGB*). The spinal cord samples also show consistently strong membership in a single cluster (yellow-orange), the top defining gene for the cluster being *MBP* which is involved in myelination of nerves in the nervous system [27]. Another driving gene, *GFAP*, participates in system development by acting as a marker to distinguish astrocytes during development [28].

Table 2. Cluster Annotations for GTEx V6 Brain data.

Cluster	Top 5 Driving Genes	Top significant GO terms
1. Royal blue	*CLU, OXT, GLUL, NDRG2, CST3*	GO:0043230 (extracellular organelle)[5e-11], GO:1903561 (extracellular vesicle)[6e-11], GO:0070062 (extracellular exosome)[2e-09], GO:0006950 (response to stress)[3e-10], GO:0031988 (membrane bound vesicle)[1e-10]
2. Turquoise	*ENC1, NCALD, YWHAH, KIF5A, NPTXR*	GO:0097458 (neuron part)[3e-11], GO:0008092 (cytoskeletal protein binding)[7e-11], GO:0031175 (neuron projection development)[7e-09], GO:0030182 (neuron differentiation)[4e-08], GO:0007268 (synaptic transmission)[1e-08]
3. Lime green	*PKD1, CBLN3, CHGB, COL27A1, ABLIM1*	GO:0005089 (Rho guanyl-nucleotide exchange factor activity)[1e-03], GO:0016604 (nuclear body)[0.002], GO:0022008 (neurogenesis)[0.02], GO:0035239 (tube morphogenesis)[0.08], GO:0007269 (neurotransmitter secretion)[0.10]
4. Red	*PPP1R1B, RGS14, NCDN, PDE1B, RAP1GAP*	GO:0065009 (regulation of molecular function)[2e-06], GO:0036477 (somatodendritic compartment)[6e-05], GO:0007268 (synaptic transmission)[1e-03], GO:0023051 (signaling regulation)[2e-03], GO:0010646 (cell communication regulation)[1e-03]
5. Yellow orange	*MBP, GFAP, TF, MTURN, SCD*	GO:0043209 (myelin sheath)[2e-09], GO:0007399 (nervous system development)[1e-04], GO:0005737 (cytoplasm)[1e-04], GO:0048471 (perinuclear region of cytoplasm)[5e-04], GO:0007272 (ensheathment of neurons)[1e-02]
6. Yellow	*IQGAP1, A2M, C3, MYH7, TG*	GO:0072562 (blood microparticle)[1e-10], GO:0044449 (contractile fiber part)[1e-10], GO:0043230 (extracellular organelle)[7e-10], GO:0030017 (sarcomere)[1e-08], GO:0072376 (protein activation cascade)[1e-08]

doi:10.1371/journal.pgen.1006599.t002

The remaining samples all show membership in multiple clusters. Samples from the putamen, caudate and nucleus accumbens show similar profiles, and are distinguished by strong membership in a cluster (cluster 4, bright red) whose top driving gene is *PPP1R1B*, a target for dopamine. And cortex samples are distinguished from others by stronger membership in a cluster (cluster 2, turquoise in Fig 1(b)) whose distinctive genes include *ENC1*, which interacts with actin and contributes to the organisation of the cytoskeleton during the specification of neural fate [29].

In comparison, applying PCA, MDS, hierarchical clustering and *t*-SNE to these brain samples reveals less of this finer-scale structure (S4 Fig). Both PCA and MDS effectively cluster the samples into two groups—those related to the cerebellum vs everything else. Hierarchical clustering also separates out the cerebellum-related tissues from the others, but again the format seems ill-suited to static visualization of more than one thousand samples. For reasons that we do not understand *t*-SNE performs poorly for these data: many samples are allocated to essentially identical locations, and so overplotting obscures them.

Single-cell RNA-seq data

Recently RNA-sequencing has become viable for single cells [30], and this technology has the promise to revolutionize understanding of intra-cellular variation in expression, and regulation more generally [31]. Although it is traditional to describe and categorize cells in terms of distinct cell-types, the actual architecture of cell heterogeneity may be more complex, and in some cases perhaps better captured by the more "continuous" GoM model. In this section we illustrate the potential for the GoM model to be applied to single cell data.

To be applicable to single-cell RNA-seq data, methods must be able to deal with lower sequencing depth than in bulk RNA experiments: single-cell RNA-seq data typically involve substantially lower effective sequencing depth compared with bulk experiments, due to the relatively small number of molecules available to sequence in a single cell. Therefore, as a first step towards demonstrating its potential for single cell analysis, we checked robustness of the GoM model to sequencing depth. Specifically, we repeated the analyses above after thinning the GTEx data by a factor of 100 and 10,000 to mimic the lower sequencing depth of a typical single cell experiment. For the thinned GTEx data the Structure plot for $K = 20$ preserves most of the major features of the original analysis on unthinned data (S5 Fig). For the accuracy comparisons with hierarchical clustering, both methods suffer reduced accuracy in thinned data, but the GoM model remains superior (S6 Fig). For example, when thinning by a factor of 10,000, the success rate in separating pairs of tissues is 0.32 for the GoM model vs 0.10 for hierarchical clustering.

Having established its robustness to sequencing depth, we now illustrate the GoM model on two single cell RNA-seq datasets: data on mouse spleen from Jaitin *et al* [32] and data on mouse preimplantation embryos from Deng *et al* [33].

Mouse spleen data from Jaitin et al, 2014. Jaitin *et al* sequenced over 4,000 single cells from mouse spleen. Here we analyze 1,041 of these cells that were categorized as *CD*11*c*+ in the *sorting markers* column of their data (http://compgenomics.weizmann.ac.il/tanay/?page_id=519), and which had total number of reads mapping to non-ERCC genes greater than 600. Our hope was that applying the GoM model to these data would identify, and perhaps refine, the cluster structure evident in [32] (their Fig 2A and 2B). However, the GoM model yielded rather different results (Fig 3), where most cells were assigned to have membership in several clusters. Further, the cluster membership vectors showed systematic differences among amplification batches (which in these data is also strongly correlated with sequencing batch). For example, cells in batch 1 are characterized by strong membership in the orange cluster (cluster

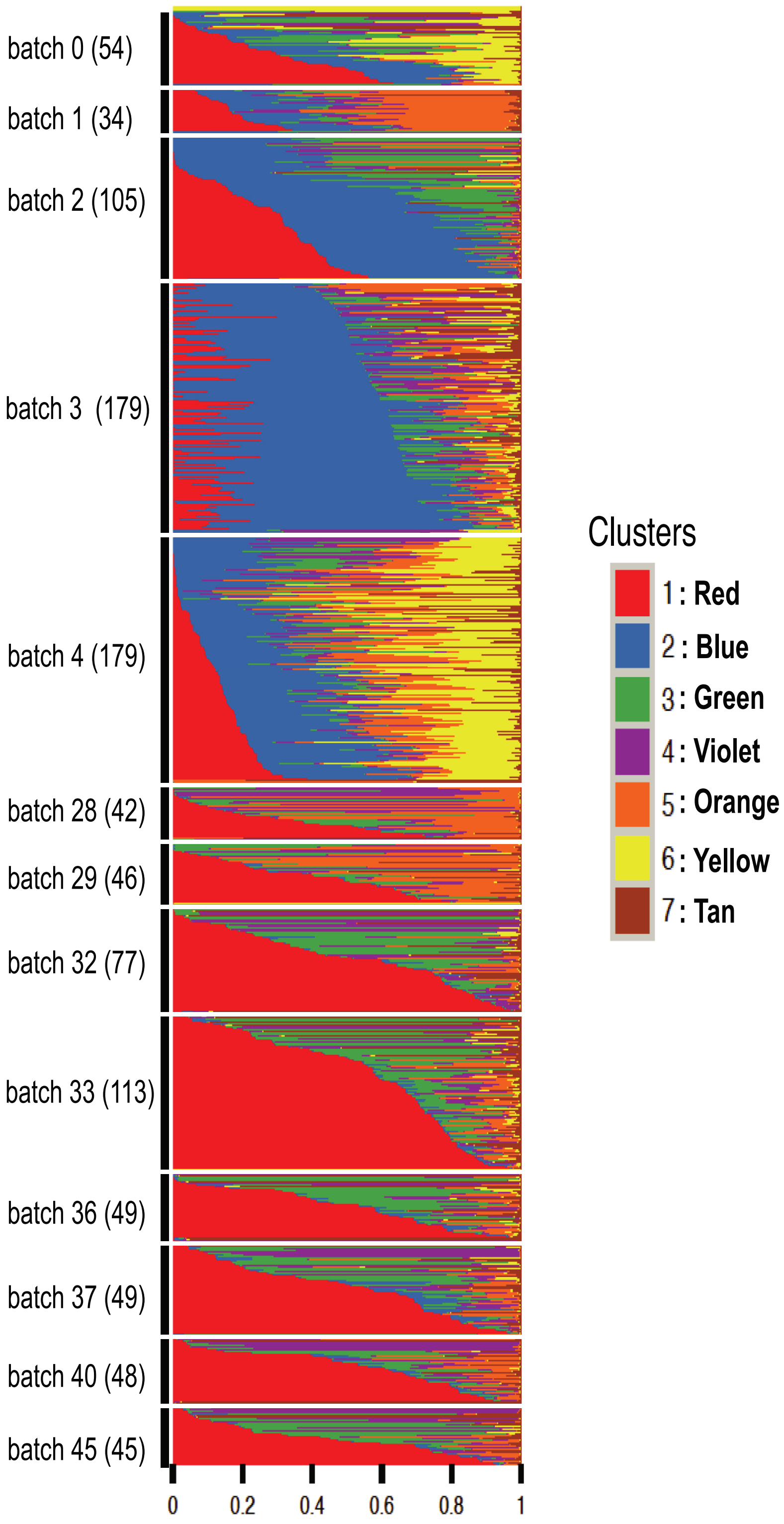

Fig 3. Structure plot of estimated membership proportions for GoM model with K = 7 clusters fit to 1,041 single cells from [33]. The samples (cells) are ordered so that samples from the same amplification batch are adjacent and within each batch, the samples are sorted by the proportional representation of the underlying clusters. In this analysis the samples do not appear to form clearly-defined clusters, with each sample being allocated membership in several "clusters". Membership proportions are correlated with batch, and some groups of batches (e.g. 28–29; 32–45) show similar palettes. These results suggest that batch effects are likely influencing the inferred structure in these data.

doi:10.1371/journal.pgen.1006599.g003

5) while those in batch 4 are characterized by strong membership in both the blue and yellow clusters (2 and 6). Some adjacent batches show similar patterns—for example batches 28 and 29 have a similar visual "palette", as do batches 32–45. And, more generally, these later batches are collectively more similar to one another than they are to the earlier batches (0–4).

The fact that batch effects are detectable in these data is not particularly surprising: there is a growing recognition of the importance of batch effects in high-throughput data generally [34, 35] and in single cell data specifically [26, 36]. And indeed, both clustering methods and the GoM model can be viewed as dimension reduction methods, and such methods can be helpful in controlling for batch effects [37, 38]. However, why these batch effects are not evident in Fig 2A and 2B of [32] is unclear.

Mouse preimplantation embryo data from Deng et al, 2014. Deng *et al* collected single-cell expression data of mouse preimplantation embryos from the zygote to blastocyst stage [33], with cells from four different embryos sequenced at each stage. The original analysis [33] focuses on trends of allele-specific expression in early embryonic development. Here we use the GoM model to assess the primary structure in these data without regard to allele-specific effects (i.e. combining counts of the two alleles). Visual inspection of the Principal Components Analysis in [33] suggested perhaps 6–7 clusters, and we focus here on results with $K = 6$.

The results from the GoM model (Fig 4) clearly highlight changes in expression profiles that occur through early embryonic development stages, and enrichment analysis of the driving genes in each cluster (Table 3, S4 Table) indicate that many of these expression changes reflect important biological processes during embryonic preimplantation development.

In more detail: Initially, at the zygote and early 2-cell stages, the embryos are represented by a single cluster (blue in Fig 4) that is enriched with genes responsible for germ cell development (e.g., *Bcl2l10* [39], *Spin1* [40]). Moving through subsequent stages the grades of membership evolve to a mixture of blue and magenta clusters (mid 2-cell), a mixture of magenta and yellow clusters (late 2-cell) and a mixture of yellow and green (4-cell stage). The green cluster then becomes more prominent in the 8-cell and 16-cell stages, before dropping substantially in the early and mid-blastocyst stages. That is, we see a progression in the importance of different clusters through these stages, from the blue cluster, moving through magenta and yellow to green. Examining the genes distinguishing each cluster reveals that this progression (blue-magenta-yellow-green) reflects the changing relative importance of several fundamental biological processes. The magenta cluster is driven by genes responsible for the beginning of transcription of zygotic genes (e.g., *Zscan4c-f* show up in the list of top 100 driving genes: see https://stephenslab.github.io/count-clustering/project/src/deng_cluster_annotations.html), which takes place in the late 2-cell stage of early mouse embryonic development [41]. The yellow cluster is enriched for genes responsible for heterochromation *Smarcc1* [42] and chromosome stability *Cenpe* [43] (see S4 Table). And the green cluster is enriched for cytoskeletal genes (e.g., *Fbxo15*) and cytoplasm genes (e.g., *Tceb1*, *Hsp90ab1*), all of which are essential for compaction at the 8-cell stage and morula formation at the 16-cell stage.

Finally, during the blastocyst stages two new clusters (purple and orange in Fig 4) dominate. The orange cluster is enriched with genes involved in the formation of trophectoderm (TE) (e.g., *Tspan8*, *Krt8*, *Id2* [44]), while the purple cluster is enriched with genes responsible for the formation of inner cell mass (ICM) (e.g., *Pdgfra*, *Pyy* [45]).

For comparison, results for PCA, MDS, *t*-SNE and hierarchical clustering are shown in S7 Fig. All these methods show some clustering structure by pre-implantation stage; however only PCA and MDS seem to capture the developmental trajectory from zygote to blastocyst, exhibiting a "horse-shoe" pattern that is expected when similarities among samples approximately reflect an underlying latent ordering [46, 47]. And none of them provide any direct indication of the ICM vs TE structure in the blastocyst samples.

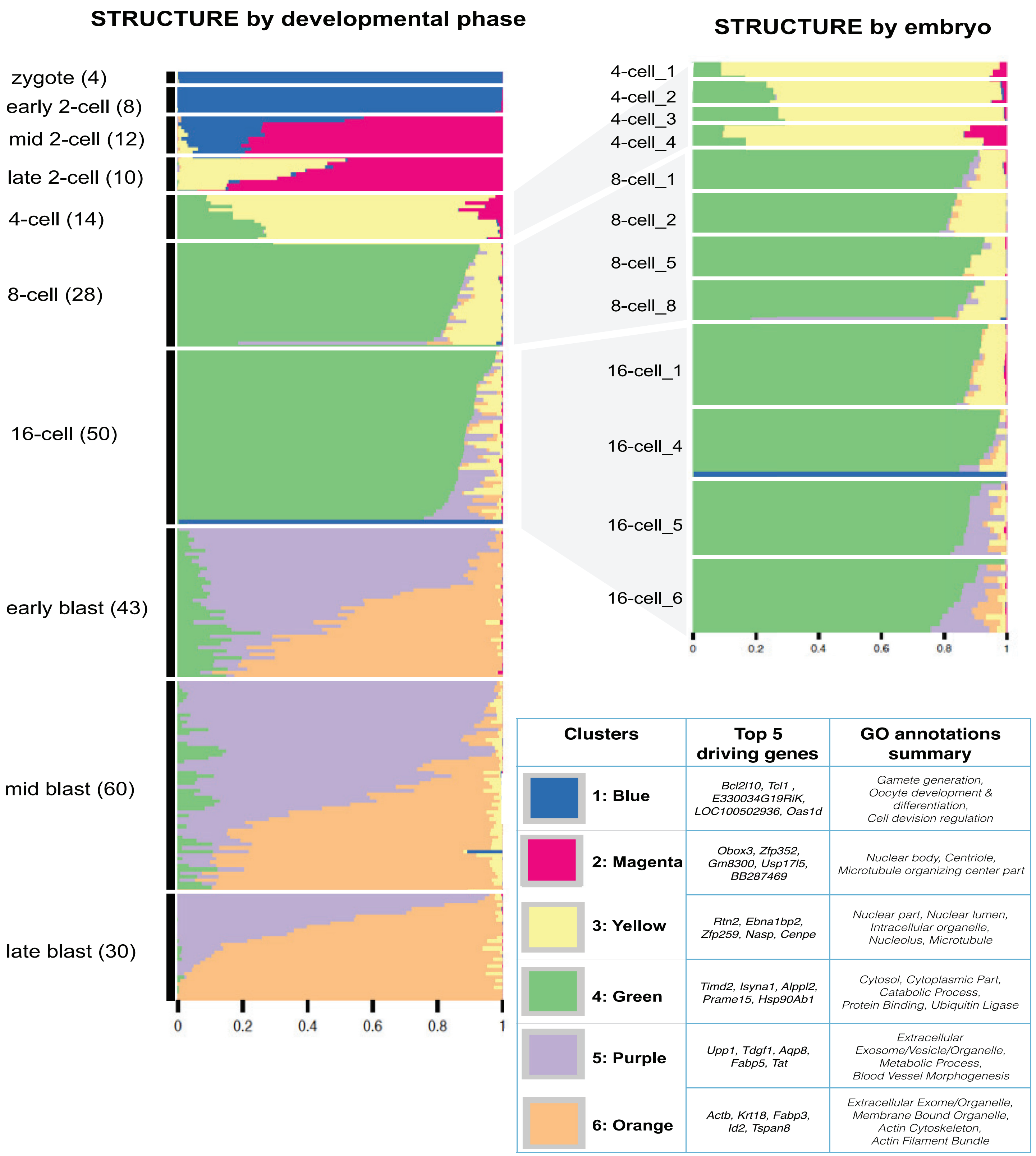

Clusters	Top 5 driving genes	GO annotations summary
1: Blue	*Bcl2l10, Tcl1 , E330034G19RiK, LOC100502936, Oas1d*	*Gamete generation, Oocyte development & differentiation, Cell devision regulation*
2: Magenta	*Obox3, Zfp352, Gm8300, Usp17l5, BB287469*	*Nuclear body, Centriole, Microtubule organizing center part*
3: Yellow	*Rtn2, Ebna1bp2, Zfp259, Nasp, Cenpe*	*Nuclear part, Nuclear lumen, Intracellular organelle, Nucleolus, Microtubule*
4: Green	*Timd2, Isyna1, Alppl2, Prame15, Hsp90Ab1*	*Cytosol, Cytoplasmic Part, Catabolic Process, Protein Binding, Ubiquitin Ligase*
5: Purple	*Upp1, Tdgf1, Aqp8, Fabp5, Tat*	*Extracellular Exosome/Vesicle/Organelle, Metabolic Process, Blood Vessel Morphogenesis*
6: Orange	*Actb, Krt18, Fabp3, Id2, Tspan8*	*Extracellular Exome/Organelle, Membrane Bound Organelle, Actin Cytoskeleton, Actin Filament Bundle*

Fig 4. Structure plot of estimated membership proportions for GoM model with K = 6 clusters fit to 259 single cells from [33]. The cells are ordered by their preimplantation development phase (and within each phase, sorted by the proportional representation of the clusters). While the very earliest developmental phases (zygote and early 2-cell) are essentially assigned to a single cluster, others have membership in multiple clusters. Each cluster is

annotated by the genes that are most distinctively expressed in that cluster, and by the gene ontology categories for which these distinctive genes are most enriched (see Table 1 for more extensive annotation results). See text for discussion of biological processes driving these results.

doi:10.1371/journal.pgen.1006599.g004

Although the GoM model results clearly highlight some of the key biological processes underlying embryonic preimplantation development, there are also some expected patterns that do not appear. Specifically, just prior to implantation the embryo consists of three different cell types, the trophectoderm (TE), the primitive endoderm (PE), and the epiblast (EPI) [48], with the PE and EPI being formed from the ICM. Thus one might expect the late blastocyst cells to show a clear division into three distinct groups, and for some of the earlier blastocyst cells to show partial membership in one of these groups as they begin to differentiate towards these cell types. Indeed, the GoM model seems well suited to capture this process in principle. However, this is not the result we obtained in practice. In particular, although the two clusters identified by the GoM model in the blastocyst stages appear to correspond roughly to the TE and ICM, even the late blastocyst cells tend to show a gradient of memberships in both these clusters, rather than a clear division into distinct groups. Our results contrast with those from the single-cell mouse preimplantation data of [44], measured by qPCR, where the late blastocyst cells showed a clear visual division into three groups using PCA (their Fig 1).

To better understand the differences between our results for RNA-seq data from [33] and the qPCR results from [44] we applied the GoM model with $K = 3$ to a small subset of the RNA-seq data: the blastocyst cell data at the 48 genes assayed by [44]. These genes were specifically chosen by them to help elucidate cell-fate decisions during early development of the mouse embryo. Still, the GoM model results (S8 Fig) do not support a clear division of these data into three distinct groups (and neither do PCA or *t*-SNE; S9 Fig). Rather, the GoM model highlights one cluster (Green in figure), whose membership proportions essentially reflect expression at the *Actb* gene, and two other clusters (Orange and Purple in figure) whose

Table 3. Cluster Annotations for Deng et al (2014) data.

Cluster	Top 10 Driving Genes	Top significant GO terms
1. Blue	*Bcl2l10*, *E330034G19Rik*, *Tcl1*, *LOC100502936*, *Oas1d*, *AU022751*, *Spin1*, *Khdc1b*, *D6Ertd527e*, *Btg4*	GO:0007276 (gamete generation)[7e-06], GO:0032504 (multicellular organism reproduction)[3e-06], GO:0044702 (single organism reproduction)[2e-05], GO:0048477 (oogenesis)[5e-04], GO:0048599 (oocyte development)[1e-03], GO:0009994 (oocyte differentiation)[1e-03]
2. Magenta	*Obox3*, *Zfp352*, *Gm8300*, *Usp17l5*, *BB287469*, *Rfpl4b*, *Gm2022*, *Gm5662*, *Gm11544*, *Gm4850*	GO:0016604 (nuclear body)[1e-04], GO:0005814 (centriole)[4e-03], GO:0044450 (microtubule organizing center part) [8e-03]
3. Yellow	*Rtn2*, *Ebna1bp2*, *Zfp259*, *Nasp*, *Cenpe*, *Rnf216*, *Ctsl*, *Tor1b*, *Ankrd10*, *Lamp2*	GO:0044428 (nuclear part)[1e-08], GO:0031981 (nuclear lumen)[3e-08], GO:0070013 (intracellular organelle lumen)[9e-08], GO:0005730 (nucleolus)[5e-07], GO:0005654 (nucleoplasm)[4e-05], GO:0003723 (RNA binding)[1e-04]
4. Green	*Timd2*, *Isyna1*, *Alppl2*, *Prame15*, *Fbxo15*, *Tceb1*, *Gpd1l*, *Pemt*, *Hsp90aa1*, *Hsp90ab1*	GO:0005829 (cytosol)[4e-10], GO:0044444 (cytoplasmic part)[2e-05], GO:1901575 (organic substance catabolic process)[9e-04], GO:0000151 (ubiquitin ligase com- plex)[1e-04], GO:0009056 (catabolic process)[1e-03], GO:0044265 (cellular macromolecule catabolic process)[1e-03], GO:0051082 (unfolded protein binding)[9e-04]
5. Purple	*Upp1*, *Tdgf1*, *Aqp8*, *Fabp5*, *Tat*, *Pdgfra*, *Pyy*, *Prdx1*, *Col4a1*, *Spp1*	GO:0044710 (single-organism metabolic process) [1e-05], GO:0006950 (response to stress) [1e-05], GO:0070062 (extracellular exosome)[1e-05], GO:0043230 (extracellular organelle)[2e-05], GO:1903561 (extracellular vesicle) [1e-05], GO:0006979 (response to oxidative stress)[7e-04], GO:0048514 (blood vessel morphogenesis)[7e-04], GO:0001944 (vasculature development)[3e-03]
6. Orange	*Actb*, *Krt18*, *Fabp3*, *Id2*, *Tspan8*, *Gm2a*, *Lgals1*, *Adh1*, *Lrp2*, *BC051665*	GO:0065010 (extracellular membrane-bounded organelle), GO:0070062 (extracellular exosome)[4e-23], GO:0043230 (extracellular organelle)[5e-23], GO:1903561 (extracellular vesicle)[3e-23], GO:0031982 (vesicle)[4e-18], GO:0030036 (actin cytoskeleton and organization)[4e-12], GO:0032432 (actin filament bundle)[2e-09], GO:0005912 (adherens junction)[2e-09]

doi:10.1371/journal.pgen.1006599.t003

driving genes correspond to genes identified in [44] as being distinctive to TE and EPI cell types respectively. The *Actb* gene is a "housekeeping gene", used by [44] to normalize their qPCR data, and its prominence in the GoM results likely reflects its very high expression levels relative to other genes. However, excluding *Actb* from the analysis still does not lead to a clear separation into three groups (S8 Fig). Thus, although there are clear commonalities in the structure of these RNA-seq and qPCR data sets, the structure of the single-cell RNA-seq data from [33] is fundamentally more complex (or, perhaps, muddied), and consequently more difficult to interpret.

In addition to trends across development stages, the GoM results also highlight some embryo-level effects in the early stages (Fig 4). Specifically, cells from the same embryo sometimes show greater similarity than cells from different embryos. For example, while all cells from the 16-cell stage have high memberships in the green cluster, cells from two of the embryos at this stage have memberships in both the purple and yellow clusters, while the other two embryos have memberships only in the yellow cluster.

The GoM results also highlight a few single cells as outliers. For example, a cell from a 16-cell embryo is represented by the blue cluster—a cluster that represents cells at the zygote and early 2-cell stage. Also, a cell from an 8-stage embryo has strong membership in the purple cluster—a cluster that represents cells from the blastocyst stage. This illustrates the potential for the GoM model to help in quality control: it would seem prudent to consider excluding these outlier cells from subsequent analyses of these data.

Discussion

Our goal here is to highlight the potential for GoM models to elucidate structure in RNA-seq data from both single cell sequencing and bulk sequencing of pooled cells. We also provide tools to identify which genes are most distinctively expressed in each cluster, to aid interpretation of results. As our applications illustrate, the results can provide a richer summary of the structure in RNA-seq data than existing widely-used visualization methods such as PCA and hierarchical clustering. While it could be argued that the GoM model results sometimes raise more questions than they answer, this is exactly the point of an exploratory analysis tool: to highlight issues for investigation, identify anomalies, and generate hypotheses for future testing.

Our results from different methods also highlight another important point: different methods have different strengths and weaknesses, and can compliment one another as well as competing. For example, *t*-SNE seems to provide a much clearer indication of the cluster structure in the full GTEx data than does PCA, but does a poorer job of capturing the ordering of the developmental samples from mouse pre-implantation embryos. While we believe the GoM model often provides a richer summary of the sample structure, we would expect to use it in addition to *t*-SNE and PCA when performing exploratory analyses. (Indeed the methods can be used in combination: both PCA and *t*-SNE can be used to visualize the results of the GoM model, as an alternative or complement to the Structure plot.)

A key feature of the GoM model is that it allows that each sample has a proportion of membership in each cluster, rather than a discrete cluster structure. Consequently it can provide insights into how well a particular dataset really fits a "discrete cluster" model. For example, consider the results for the data from Jaitin *et al* [32] and Deng *et al* [33]: in both cases most samples are assigned to multiple clusters, although the results are closer to "discrete" for the latter than the former. The GoM model is also better able to represent the situation where there is not really a single clustering of the samples, but where samples may cluster differently at different genes. For example, in the GTEx data, the stomach samples share memberships in

common with both the pancreas (purple) and the adrenal gland (light green). This pattern can be seen in the Structure plot (S4 Fig) but not from other methods like PCA, *t*-SNE or hierarchical clustering (Fig 2).

Fitting GoM models can be computationally-intensive for large data sets. For the datasets we considered here the computation time ranged from 12 minutes for the data from [33] ($n = 259; K = 6$), through 33 minutes for the data from [32] ($n = 1{,}041; K = 7$) to 3,370 minutes for the GTEx data ($n = 8{,}555; K = 20$). Computation time can be reduced by fitting the model to only the most highly expressed genes, and we often use this strategy to get quick initial results for a dataset. Because these methods are widely used for clustering very large document datasets there is considerable ongoing interest in computational speed-ups for very large datasets, with "on-line" (sequential) approaches capable of dealing with millions of documents [49] that could be useful in the future for very large RNA-seq datasets.

A thorny issue that arises when fitting clustering models is how to select the number of clusters, K. Like many software packages for fitting these models, the `maptpx` package implements a measure of model fit that provides one useful guide. However, it is worth remembering that in practice there is unlikely to be a "true" value of K, and results from different values of K may complement one another rather than merely competing with one another. For example, seeing how the fitted model evolves as K increases is one way to capture some notion of hierarchy in the clusters identified [21]. More generally it is often fruitful to analyse data in multiple ways using the same tool: for example our GTEx analyses illustrate how analysis of subsets of the data (in this case the brain samples) can complement analyses of the entire data. Finally, as a practical matter, we note that Structure plots can be difficult to read for large K (e.g. $K = 30$) because of the difficulties of choosing a palette with K distinguishable colors.

The version of the GoM model fitted here is relatively simple, and could certainly be embellished. For example, the model allows the expression of each gene in each cluster to be a free parameter, whereas we might expect expression of most genes to be "similar" across clusters. This is analogous to the idea in population genetics applications that allele frequencies in different populations may be similar to one another [50], or in document clustering applications that most words may not differ appreciably in frequency in different topics. In population genetics applications incorporating this idea into the model, by using a correlated prior distribution on these frequencies, can help improve identification of subtle structure [50] and we would expect the same to happen here for RNA-seq data.

Finally, GoM models can be viewed as one of a larger class of "matrix factorization" approaches to understanding structure in data, which also includes PCA, non-negative matrix factorization (NMF), and sparse factor analysis (SFA); see [51]. This observation raises the question of whether methods like SFA might be useful for the kinds of analyses we performed here. (NMF is so closely related to the GoM model that we do not discuss it further; indeed, the GoM model is a type of NMF, because both grades of membership and expression levels within each cluster are required to be non-negative.) Informally, SFA can be thought of as a generalization of the GoM model that allows samples to have *negative* memberships in some "clusters" (actually, "factors"). This additional flexibility should allow SFA to capture certain patterns more easily than the GoM model. For example, a small subset of genes that are over-expressed in some samples and under-expressed in other samples could be captured by a single sparse factor, with positive loadings in the over-expressed samples and negative loadings in the other samples. However, this additional flexibility also comes at a cost of additional complexity in visualizing the results. For example, S10, S11 and S12 Figs show results of SFA (the version from [51]) for the GTEx data and the mouse preimplantation data: in our opinion, these do not have the simplicity and immediate visual appeal of the GoM model results. Also, applying SFA to RNA-seq data requires several decisions to be made that can greatly impact the results:

what transformation of the data to use; what method to induce sparsity (there are many; e.g. [51–54]); whether to induce sparsity in loadings, factors, or both; etc. Nonetheless, we certainly view SFA as complementing the GoM model as a promising tool for investigating the structure of RNA-seq data, and as a promising area for further work.

Materials and methods

Model fitting

We use the `maptpx` R package [20] to fit the GoM models (1 and 2), which is also known as "Latent Dirichlet Allocation" (LDA). The `maptpx` package fits this model using an EM algorithm to perform Maximum a posteriori (MAP) estimation of the parameters q and θ. See [20] for details.

Visualizing results

In addition to the Structure plot, we have also found it useful to visualize results using t-distributed Stochastic Neighbor Embedding (t-SNE), which is a method for visualizing high dimensional datasets by placing them in a two dimensional space, attempting to preserve the relative distance between nearby samples [24, 25]. Compared with the Structure plot our t-SNE plots contain less information, but can better emphasize clustering of samples that have similar membership proportions in many clusters. Specifically, t-SNE tends to place samples with similar membership proportions together in the two-dimensional plot, forming visual "clusters" that can be identified by eye (e.g. http://stephenslab.github.io/count-clustering/project/src/tissues_tSNE_2.html). This may be particularly helpful in settings where no external information is available to aid in making an informative Structure plot.

Cluster annotation

To help biologically interpret the clusters, we developed a method to identify which genes are most distinctively differentially expressed in each cluster. (This is analogous to identifying "ancestry informative markers" in population genetics applications [55].) Specifically, for each cluster k we measure the distinctiveness of gene g with respect to any other cluster l using

$$\mathrm{KL}^{g}[k, l] := \theta_{kg}\ log\frac{\theta_{kg}}{\theta_{lg}} + \theta_{lg} - \theta_{kg}, \tag{3}$$

which is the Kullback–Leibler divergence of the Poisson distribution with parameter θ_{kg} to the Poisson distribution with parameter θ_{lg}. For each cluster k, we then define the distinctiveness of gene g as

$$D^{g}[k] = \min_{l \neq k}\ \mathrm{KL}^{g}[k, l]. \tag{4}$$

The higher $D^g[k]$, the larger the role of gene g in distinguishing cluster k from all other clusters. Thus, for each cluster k we identify the genes with highest $D^g[k]$ as the genes driving the cluster k. We annotate the biological functions of these individual genes using the `mygene` R Bioconductor package [56].

For each cluster k, we filter out a number of genes (top 100 for the Deng *et al* data [33] and GTEx V6 data [57]) with highest $D^g[k]$ value and perform a gene set over-representation analysis of these genes against all the other genes in the data representing the background. To do this, we used ConsensusPathDB database (http://cpdb.molgen.mpg.de/) [58] [59]. See Tables

1, 2 and 3 for the top significant gene ontologies driving each cluster in the GTEx V6 data and the Deng *et al* data respectively.

Comparison with hierarchical clustering

We compared the GoM model with a distance-based hierarchical clustering algorithm by applying both methods to samples from pairs of tissues from the GTEx project, and assessed their accuracy in separating samples according to tissue. For each pair of tissues we randomly selected 50 samples from the pool of all samples coming from these tissues. For the hierarchical clustering approach we cut the dendrogram at $K = 2$, and checked whether or not this cut partitions the samples into the two tissue groups. (We applied hierarchical clustering using Euclidean distance, with both complete and average linkage; results were similar and so we showed results only for complete linkage.)

For the GoM model we analysed the data with $K = 2$, and sorted the samples by their membership in cluster 1. We then partitioned the samples at the point of the steepest fall in this membership, and again we checked whether this cut partitions the samples into the two tissue groups. S3 Fig shows, for each pair of tissues, whether each method successfully partitioned the samples into the two tissue groups.

Thinning

We used "thinning" to simulate lower-coverage data from the original higher-coverage data. Specifically, if c_{ng} is the counts of number of reads mapping to gene g for sample n for the original data, we simulated thinned counts t_{ng} using

$$t_{ng} \sim Bin(c_{ng}, p_{thin}) \tag{5}$$

where p_{thin} is a specified thinning parameter.

Code availability

Our methods are implemented in an R package `CountClust`, available as part of the Bioconductor project at https://www.bioconductor.org/packages/3.3/bioc/html/CountClust.html. The development version of the package is also available at https://github.com/kkdey/CountClust.

Code for reproducing results reported here is available at http://stephenslab.github.io/count-clustering/.

Supporting information

S1 Fig. Structure plot of GTEx V6 tissue samples for (a) $K = 5$, (b) $K = 10$, (c) $K = 15$, (d) $K = 20$. Some tissues form a separate cluster from the other tissues from $K = 5$ onwards (for example: Whole Blood, Skin), whereas some tissue only form a distinctive subgroup at $K = 20$ (for example: Arteries).
(PDF)

S2 Fig. Top five principal components (PC) for GTEx V6 tissue samples. Scatter plot representation of the top five PCs of the GTEx tissue samples. Data was transformed to log2 counts per million (CPM).
(PDF)

S3 Fig. Comparison between GoM model and hierarchical clustering under different scenarios of data transformation. We used GTEx V6 data for model performance comparisons.

Specifically, for every pair of the 53 tissues, we assessed the ability of the methods to separate samples according to their tissue of origin. The subplots of heatmaps show the results of evaluation under different scenarios. Filled squares in the heatmap indicate successful separation of the samples in corresponding tissue pair comparison. (a) Hierarchical clustering on log2 counts per million (CPM) transformed data using Euclidean distance. (b) Hierarchical clustering on the standardized log2-CPM transformed data (transformed values for each gene was mean and scale transformed) using the Euclidean distance. (c) GoM model of $K = 2$ applied to counts. (d) Hierarchical clustering on counts data with the assumption that, for each gene the sample read count c_{ng} has a variance $\bar{c}_g + 1$ that is constant across samples. And, the the gene-specific variance $\bar{c}_g + 1$ was used to scale the distance matrix for clustering. (e) Hierarchical clustering applied to adjusted count data. Each gene has a mean expression value of 0 and variance of 1. Taken together, these results suggest that regardless of the different data transformation scenarios, the GoM model with $K = 2$ is able to separate samples of different tissue of origin, better than hierarchical cluster methods.
(PDF)

S4 Fig. GTEx brain PCA, t-SNE and MDS.
(PDF)

S5 Fig. Structure plot of GTEx V6 tissue samples for $K = 20$ in two runs under different thinning parameter settings. (a) $p_{thin} = 0.01$ and (B) $p_{thin} = 0.0001$. The structure in these two plots closely resemble the pattern observed in Fig 1(a), though there are a few differences from the unthinned version.
(PDF)

S6 Fig. A comparison of accuracy of hierarchical clustering vs GoM on thinned GTEx data, with thinning parameters of $p_{thin} = 0.01$ and $p_{thin} = 0.001$. For each pair of tissue samples from the GTEx V6 data we assessed whether or not each clustering method (with $K = 2$ clusters) separated the samples according to their tissue of origin, with successful separation indicated by a filled square. Thinning deteriorates accuracy compared with the unthinned data (Fig 2), but even then the model-based method remains more successful than the hierarchical clustering in separating the samples by tissue or origin.
(PDF)

S7 Fig. Deng et al (2014) PCA, tSNE, MDS and dendrogram plots for hierarchical clustering.
(PDF)

S8 Fig. Additional GoM analysis of Deng et al (2014) data including blastocyst samples and 48 blastocyst marker genes. We considered 48 blastocyst marker genes (as chosen by Guo et al., 2010) and fitted GoM model with $K = 3$ to 133 blastocyst samples. In the Structure plot, blastocyst samples are arranged in order of estimated membership proportion in the Green cluster. The panel located above the Structure plot shows the corresponding pre-implantation stage from which blastocyst samples were collected. The heatmap located below the Structure plot represents expression levels of the 48 blastocyst marker genes (log2 CPM), and the corresponding dendrogram shows results of hierarchical clustering (complete linkage). The table on the right of the expression heatmap displays gene information, showing, from left to right, 1) whether or not the gene is a transcription factor, 2) the driving GoM cluster if the gene was among the top five driving genes, and 3) the featured cell type (TE: trophecoderm, EPI: epiblast, PE: primitive endoderm) that was found in Guo et al., 2010.
(PDF)

S9 Fig. Visualization of PCA and t-SNE results of mouse pre-implantation embryos data from Deng et al (2014) using 48 blastocyst marker genes.
(PDF)

S10 Fig. Sparse Factor Analysis loadings visualization of GTEx V6 tissue samples. The colors represent the 20 different factors. The factor loadings are presented in a stacked bar for each sample. We performed SFA under the scenarios of when the loadings are sparse (left panel) and when the factors are sparse (right panel).
(PDF)

S11 Fig. Sparse Factor Analysis loadings visualization of GTEx brain tissue samples. The colors represent the 6 different factors. The factor loadings are presented in a stacked bar for each sample. We performed SFA under the scenarios of when the loadings are sparse (left panel) and when the factors are sparse (right panel).
(PDF)

S12 Fig. Sparse Factor Analysis loadings visualization of mouse pre-implantation embryos from Deng et al., (2014). The colors represent the 6 different factors. The factor loadings are presented in a stacked bar for each sample. We performed SFA under the scenarios of when the loadings are sparse (left panel) and when the factors are sparse (right panel).
(PDF)

S1 Table. Cluster Annotations of GTEx V6 data with top driving gene summaries.
(PDF)

S2 Table. Cluster Annotations of GTEx V6 Brain data with top driving gene summaries.
(PDF)

S3 Table. Cluster Annotations of Deng data with top driving genes.
(PDF)

S4 Table. Cluster Annotation of Deng data analysis using 48 genes with top driving gene summaries.
(PDF)

Acknowledgments

The GTEx data used for the analyses described in this paper were obtained from the GTEx Portal on 10/19/2015, dbGaP Study Accession phs000424.v6.p1. We thank Matt Taddy, Amos Tanay, Effi Kenigsberg, Yoav Gilad and Jonathan Pritchard for helpful discussions. We thank Po-Yuan Tung and John Blischak for helpful comments on a draft manuscript.

Author Contributions

Conceptualization: MS KKD.

Data curation: MS.

Formal analysis: KKD CJH.

Funding acquisition: MS.

Investigation: MS.

Methodology: MS KKD.

Project administration: MS.

Resources: MS.

Software: KKD CJH MS.

Supervision: MS.

Validation: KKD CJH MS.

Visualization: KKD CJH MS.

Writing – original draft: KKD CJH MS.

Writing – review & editing: KKD CJH MS.

References

1. Eisen MB, Spellman PT, Brown PO, Botstein D. Cluster analysis and display of genome-wide expression patterns. Proceedings of the National Academy of Sciences. 1998 Dec; 95(25):14863–8. doi: 10.1073/pnas.95.25.14863
2. Golub TR, Slonim DK, Tamayo P, Huard C, Gaasenbeek M, Mesirov JP, et al. Molecular classification of cancer: class discovery and class prediction by gene expression monitoring. Science. 1999 Oct; 286 (5439):531–7. doi: 10.1126/science.286.5439.531 PMID: 10521349
3. Alizadeh AA, Eisen MB, Davis RE, Ma C, Lossos IS, Rosenwald A, Boldrick JC, et al. Distinct types of diffuse large B-cell lymphoma identified by gene expression profiling. Nature. 2000; 403(6769):503–11. doi: 10.1038/35000501 PMID: 10676951
4. D'haeseleer P. How does gene expression clustering work? Nature Biotechnology. 2005; 23(12):1499–501. doi: 10.1038/nbt1205-1499 PMID: 16333293
5. Jiang D, Tang C, Zhang A. Cluster Analysis for Gene Expression Data: A Survey. IEEE Transactions on Knowledge and Data Engineering. 2004 Nov; 16(11):1370–86. doi: 10.1109/TKDE.2004.68
6. Erosheva EA. Latent class representation of the grade of membership model. Seattle (WA): Department of Statistics, University of Washington. 2006;Technical report No.:492.
7. Pritchard JK, Stephens M, Donnelly P. Inference of population structure using multilocus genotype data. Genetics. 2000 June; 155(2):945–59. PMID: 10835412
8. Blei DM, Ng AY, Jordan MI. Latent Dirichlet Allocation. Journal of Machine Learning Research. 2003 Jan; 3:993–1022.
9. Blei DM, Lafferty J. Topic Models. In: Srivastava A, Sahami M, editors. Text mining: Classification, clustering, and applications. 2009 Jun; 10(71):34.
10. Shiraishi Y, Tremmel G, Miyano S, Stephens M. A simple model-based approach to inferring and visualizing cancer mutation signatures. PLoS Genetics. 2015 Dec; 11(12):e1005657. doi: 10.1371/journal.pgen.1005657 PMID: 26630308
11. Lindsay J, Mandoiu I, Nelson C. Gene expression deconvolution using single-cells. Proceedings of the 2013 American Association of Human Genetics meeting. 2013. http://dna.engr.uconn.edu/bibtexmngr/upload/Lal.13.pdf.
12. Ahn J, Yuan Y, Parmigiani G, Suraokar MB, Diao L, Wistuba II, et al. DeMix: deconvolution for mixed cancer transcriptomes using raw measured data. Bioinformatics. 2013 Aug; 29(15):1865–71. doi: 10.1093/bioinformatics/btt301 PMID: 23712657
13. Quon G, Haider S, Deshwar AG, Cui A, Boutros PC, Morris Q. Computational purification of individual tumor gene expression profiles leads to significant improvements in prognostic prediction. Genome Medicine. 2013 Mar; 5(3):29. doi: 10.1186/gm433 PMID: 23537167
14. Repsilber D, Kern S, Telaar A, Walzl G, Black GF, Selbig J, et al. Biomarker discovery in heterogeneous tissue samples—taking the in-silico deconfounding approach. BMC Bioinformatics. 2010 Jan; 11:27. doi: 10.1186/1471-2105-11-27 PMID: 20070912
15. Schwartz R, Shackney SE. Applying unmixing to gene expression data for tumor phylogeny inference. BMC Bioinformatics. 2010 Jan; 11(1):42. doi: 10.1186/1471-2105-11-42 PMID: 20089185
16. Wang N, Gong T, Clarke R, Chen L, Shih IeM, Zhang Z, et al. UNDO: a Bioconductor R package for unsupervised deconvolution of mixed gene expressions in tumor samples. Bioinformatics. 2015 Jan; 31 (1):137–9. doi: 10.1093/bioinformatics/btu607 PMID: 25212756

17. Shen-Orr SS, Tibshirani R, Khatri P, Bodian DL, Staedtler F, Perry NM, et al. Cell type-specific gene expression differences in complex tissues. Nature Methods. 2010 Apr; 7(4):287–9. doi: 10.1038/nmeth.1439 PMID: 20208531

18. Qiao W, Quon G, Csaszar E, Yu M, Morris Q, Zandstra PW. PERT: A method for expression deconvolution of human blood samples from varied microenvironmental and developmental conditions. PLoS Computational Biology. 2012; 8(12):e1002838. doi: 10.1371/journal.pcbi.1002838 PMID: 23284283

19. Oshlack A, Robinson MD, Young MD. From RNA-seq reads to differential expression results. Genome Biology. 2010; 11(12):220. doi: 10.1186/gb-2010-11-12-220 PMID: 21176179

20. Taddy M. On Estimation and Selection for Topic Models. Proceedings of the Fifteenth International Conference on Artificial Intelligence and Statistics. 2012:1184–93.

21. Rosenberg NA, Pritchard JK, Weber JL, Cann HM, Kidd KK, Zhivotovsky LA, et al. Genetic structure of human populations. Science. 2002 Dec; 298(5602):2381–5. doi: 10.1126/science.1078311 PMID: 12493913

22. Dey K, Hsiao CJ, Stephens M. CountClust: Clustering and visualizing RNA-Seq expression data using grade of membership models. Version 1.0.2 [R/Bioconductor package]. Available from: https://www.bioconductor.org/packages/3.3/bioc/html/CountClust.html

23. Gentleman R, Carey VJ, Bates DB, Bolstad B, Dettling M, Dudoit S, et al. Bioconductor: open software development for computational biology and bioinformatics. Genome Biology. 2004 Sep; 5:R80. doi: 10.1186/gb-2004-5-10-r80 PMID: 15461798

24. Van Der Maaten L, Hinton G. Visualizing data using t-SNE. Journal of Machine Learning Research. 2008 Nov; 9:2579–2605.

25. Van Dder Maaten L. Accelerating t-SNE using tree-based algorithms. Journal of Machine Learning Research. 2015 Jan; 15:3221–3245.

26. Gilad Y, Mizrahi-Man O. A reanalysis of mouse ENCODE comparative gene expression data. Version 1. F1000Research. 2015; 4:121. doi: 10.12688/f1000research.6536.1 PMID: 26236466

27. Hu JG, Shi LL, Chen YJ, Xie XM, Zhang N, Zhu AY, et al. Differential effects of myelin basic protein-activated Th1 and Th2 cells on the local immune microenvironment of injured spinal cord. Experimental Neurology. 2016 Mar; 277:190–201. doi: 10.1016/j.expneurol.2016.01.002 PMID: 26772636

28. Baba H, Nakahira K, Morita N, Tanaka F, Akita H, Ikenaka K. GFAP gene expression during development of astrocyte. Developmental Neuroscience. 1997; 19(1):49–57. doi: 10.1159/000111185 PMID: 9078433

29. Hernandez MC, Andres-Barquin PJ, Martinez S, Bulfone A, Rubenstein JL, Israel MA. ENC-1: a novel mammalian kelch-related gene specifically expressed in the nervous system encodes an actin-binding protein. Journal of Neuroscience. 1997 May; 17(9):3038–51. PMID: 9096139

30. Tang F, Barbacioru C, Wang Y, Nordman E, Lee C, Xu N, et al. mRNA-Seq whole-transcriptome analysis of a single cell. Nature Methods. 2009 May; 6(5):377–82. doi: 10.1038/nmeth.1315 PMID: 19349980

31. Trapnell C. Defining cell types and states with single-cell genomics. Genome Research. 2015; 25:1491–8. doi: 10.1101/gr.190595.115 PMID: 26430159

32. Jaitin DA, Kenigsberg E, Keren-Shaul H, Elefant N, Paul F, Zaretsky I, et al. Massively parallel single-cell RNA-Seq for marker-free decomposition of tissues into cell types. Science. 2014 Feb; 343(6172):776–9. doi: 10.1126/science.1247651 PMID: 24531970

33. Deng Q, Ramskold D, Reinius B, Sandberg R. Single-cell RNA-Seq reveals dynamic, random monoallelic gene expression in mammalian cells. Science. 2014 Jan; 343(6167):193–6. doi: 10.1126/science.1245316 PMID: 24408435

34. Leek JT, Scharpf RB, Bravo HC, Simcha D, Langmead B, Johnson WE, et al. Tackling the widespread and critical impact of batch effects in high-throughput data. Nature Reviews Genetics. 2010 Oct; 11(10):733–9. doi: 10.1038/nrg2825 PMID: 20838408

35. Danielsson F, James T, Gomez-Cabrero D, Huss M. Assessing the consistency of public human tissue RNA-seq data sets. Briefings in Bioinformatics. 2015 Nov; 15(6):941–9. doi: 10.1093/bib/bbv017

36. Hicks SC, Teng M, Irizarry RA. On the widespread and critical impact of systematic bias and batch effects in single-cell RNA-Seq data. bioRXiv. 2015. https://doi.org/10.1101/025528

37. Leek JT, Storey JD. Capturing heterogeneity in gene expression studies by surrogate variable analysis. PLoS Genetics. 2007 Sep; 3(9):1724–35. doi: 10.1371/journal.pgen.0030161 PMID: 17907809

38. Stegle O, Parts L, Piipari M, Winn J, Durbin R. Using probabilistic estimation of expression residuals (PEER) to obtain increased power and interpretability of gene expression analyses. Nature Protocols. 2012 Feb; 7(3):500–7. doi: 10.1038/nprot.2011.457 PMID: 22343431

39. Yoon SJ, Kim EY, Kim YS, Lee HS, Kim KH, Bae J, et al. Role of Bcl2-like 10 (*Bcl2l10*) in regulating mouse oocyte maturation. Biology of Reproduction. 2009 Sep; 81(3):497–506. doi: 10.1095/biolreprod.108.073759 PMID: 19439730

40. Evsikov AV, De Evsikova C. Gene expression during the oocyte-to-embryo transition in mammals. Molecular Reproduction and Development. 2009 Sep; 76(9):805–18. doi: 10.1002/mrd.21038 PMID: 19363788

41. Falco G, Lee SL, Stanghellini I, Bassey UC, Hamatani T, Ko MS. Zscan4: a novel gene expressed exclusively in late 2-cell embryos and embryonic stem cells. Developmental Biology. 2007 Jul; 307 (2):539–50. doi: 10.1016/j.ydbio.2007.05.003 PMID: 17553482

42. Schaniel C, Ang YS, Ratnakumar K, Cormier C, James T, Bernstein E, et al. Smarcc1/Baf155 couples self-renewal gene repression with changes in chromatic structure in mouse embroynic stem cells. Stem Cells. 2009 Dec; 27(12):2979–91. PMID: 19785031

43. Putkey FR, Cramer T, Morphew MK, Silk AD, Johnson RS, McIntosh JR, et al. Unstable Kinetochore-Microtubule capture and chromosomal instability following deletion of CENP-E. Developmental Cell. 2002 Sep; 3(3):351–65. doi: 10.1016/S1534-5807(02)00255-1 PMID: 12361599

44. Guo G, Huss M, Tong GQ, Wang C, Li Sun L, Clarke ND, et al. Resolution of cell fate decisions revealed by single-cell gene expression analysis from zygote to blastocyst. Developmental Cell. 2010 Apr; 18 (4):675–85. doi: 10.1016/j.devcel.2010.02.012 PMID: 20412781

45. Hou J, Charters AM, Lee SC, Zhao Y, Wu MK, Jones SJM, et al. A systematic screen for genes expressed in definitive endoderm by Serial Analysis of Gene Expression (SAGE). BMC Developmental Biology. 2007 Aug; 7:92. doi: 10.1186/1471-213X-7-92 PMID: 17683524

46. Diaconis P, Goel S, Holmes S. Horseshoes in multidimensional scaling and local kernel methods. The Annals of Applied Statistics. 2008 Sep; 2(3):777–807. doi: 10.1214/08-AOAS165

47. Novembre J, Stephens M. Interpreting principal component analyses of spatial population genetic variation. Nature Genetics. 2008 May; 40(5):646–9. doi: 10.1038/ng.139 PMID: 18425127

48. Rossant J, Tam PP. Blastocyst lineage formation, early embryonic asymmetries and axis patterning in the mouse. Development. 2009 Mar; 136(5):701–713, doi: 10.1242/dev.017178 PMID: 19201946

49. Hoffman MD, Blei DM, Bach F. Online learning for latent Dirichlet allocation. Neural Information Processing Systems. 2010 Dec;(23):856–64.

50. Falush D, Stephens M, Pritchard JK. Inference of population structure using multilocus genotype data: linked loci and correlated allele frequencies. Genetics. 2003 Aug; 164(4):1567–87. PMID: 12930761

51. Engelhardt BE, Stephens M. Analysis of population Structure: A unifying framework and novel methods based on sparse factor analysis. PLoS Genetics. 2010 Sep; 6(9):e101117. doi: 10.1371/journal.pgen.1001117

52. Witten DM, Tibshirani R. Hastie T. A penalized matrix decomposition, with applications to sparse principal components and canonical correlation analysis. Biostatistics. 2009 May; 10(3):515–534. doi: 10.1093/biostatistics/kxp008 PMID: 19377034

53. Lopes HF, West M. Bayesian model assessment in factor analysis. Statistica Sinica. 2004 Jan; 14 (1):41–67.

54. Bhattacharya A, Dunson DB. Sparse Bayesian infinite factor models. Biometrika. 2011 Jun; 98(2):291–306. doi: 10.1093/biomet/asr013 PMID: 23049129

55. Rosenberg NA. Algorithms for selecting informative marker panels for population assignment. Journal of Computational Biology. 2005 Nov; 12(9):1183–201. doi: 10.1089/cmb.2005.12.1183 PMID: 16305328

56. Mark A, Thompson R, Afrasiabi C, Wu C. mygene: Access MyGene.Info services. Version 1.2.3 [R/Bioconductor package]. 2014. Available from: https://www.bioconductor.org/packages/release/bioc/html/mygene.html

57. GTEx Consortium. The Genotype-Tissue Expression (GTEx) project. Nature Genetics. 2013 Jun; 45 (6):580–5. doi: 10.1038/ng.2653 PMID: 23715323

58. Kamburov A, Stelzl U, Lehrach H, Herwig R. The ConsensusPathDB interaction database: 2013 update. Nucleic Acids Research. 2013 Jan; 41(Database issue):D793–800. doi: 10.1093/nar/gks1055 PMID: 23143270

59. Pentchev K, Ono K, Herwig R, Ideker T, Kamburov A. Evidence mining and novelty assessment of protein-protein interactions with the ConsensusPathDB plugin for Cytoscape. Bioinformatics. 2010 Nov; 26 (21):2796–7. doi: 10.1093/bioinformatics/btq522 PMID: 20847220
